# Oscillatory and non-oscillatory features of the magnetoencephalic sensorimotor rhythm in Parkinson’s disease

**DOI:** 10.1038/s41531-024-00669-3

**Published:** 2024-03-05

**Authors:** Mikkel C. Vinding, Josefine Waldthaler, Allison Eriksson, Cassia Low Manting, Daniel Ferreira, Martin Ingvar, Per Svenningsson, Daniel Lundqvist

**Affiliations:** 1 NatMEG, Department of Clinical Neuroscience, Karolinska Institutet, Stockholm, Sweden; 2 Danish Research Centre for Magnetic Resonance, Centre for Functional and Diagnostic Imaging and Research, Copenhagen University Hospital - Amager and Hvidovre, Copenhagen, Denmark; 3 Section of Neurology, Department of Clinical Neuroscience, Karolinska Institutet, Stockholm, Sweden; 4 Department of Neurology, University Hospital Marburg, Marburg, Germany; 5 Department of Women’s and Children’s Health, Uppsala University, Uppsala, Sweden; 6 Cognitive Neuroimaging Centre, Lee Kong Chien School of Medicine, Nanyang Technological University, Singapore, Singapore; 7 McGovern Institute of Brain Research, Massachusetts Institute of Technology, Cambridge, MA 02139 USA; 8 Division of Clinical Geriatrics, Center for Alzheimer’s Research, Department of Neurobiology, Care Sciences, and Society, Karolinska Institutet, Stockholm, Sweden; 9 Department of Radiology, Mayo Clinic, Rochester, MN USA; 10 Facultad de Ciencias de la Salud, Universidad Fernando Pessoa Canarias, Las Palmas de Gran, Canaria, España

**Keywords:** Parkinson's disease, Neuroscience

## Abstract

Parkinson’s disease (PD) is associated with changes in neural activity in the sensorimotor alpha and beta bands. Using magnetoencephalography (MEG), we investigated the role of spontaneous neuronal activity within the somatosensory cortex in a large cohort of early- to mid-stage PD patients (*N* = 78) on Parkinsonian medication and age- and sex-matched healthy controls (*N* = 60) using source reconstructed resting-state MEG. We quantified features of the time series data in terms of oscillatory alpha power and central alpha frequency, beta power and central beta frequency, and 1/f broadband characteristics using power spectral density. Furthermore, we characterised transient oscillatory burst events in the mu-beta band time-domain signals. We examined the relationship between these signal features and the patients’ disease state, symptom severity, age, sex, and cortical thickness. PD patients and healthy controls differed on PSD broadband characteristics, with PD patients showing a steeper 1/f exponential slope and higher 1/f offset. PD patients further showed a steeper age-related decrease in the burst rate. Out of all the signal features of the sensorimotor activity, the burst rate was associated with increased severity of bradykinesia, whereas the burst duration was associated with axial symptoms. Our study shows that general non-oscillatory features (broadband 1/f exponent and offset) of the sensorimotor signals are related to disease state and *oscillatory burst rate* scales with symptom severity in PD.

## Introduction

Parkinson’s disease (PD) is a common neurodegenerative disease characterised by a gradual accumulation of Lewy bodies and death of dopaminergic neurons^1,2^. The Lewy body pathology of PD begins long before the manifestation of motor symptoms. Accumulation of Lewy bodies is initially found in the olfactory bulb and brain stem and then spreads to the substantia nigra pars compacta, followed by several brain regions, including the basal ganglia and the neocortex^3^. The progressive structural and neurochemical changes in PD are accompanied by widespread functional changes in neuronal activity, which in turn lead to worsening clinical signs and symptoms such as tremor, rigidity, and bradykinesia and co-occurring non-motor symptoms like sleep disorders, depression, fatigue, and cognitive deficits^1^.

The pathophysiological changes in PD are accompanied by changes in the oscillatory activity of neurons^4^. In PD, spontaneous oscillatory beta band (13–30 Hz) activity in the subthalamic nucleus (STN) exhibits a systematic disease-related increase in synchronicity that is related to the dopamine level^5–8^, and correlates with the severity of bradykinesia and rigidity symptoms^9–11^. Changes in the beta band extend beyond the STN through the basal ganglia-thalamic cortical sensorimotor network. The cortical manifestation of the disease-related changes in the sensorimotor network can be measured non-invasively from the cortex using electro- or magnetoencephalography (EEG/MEG). Such non-invasive neural recordings can potentially provide easily available prospective biomarkers of disease or symptom-related neural changes in PD. Increased oscillatory beta-band activity in the sensorimotor cortex has been linked to increased symptom severity, such as rigidity and bradykinesia^12^. The role of dopamine on the cortical beta band is, however, still unclear. There is no consensus on how dopaminergic medication affects cortical beta-band power, with some studies reporting no effects^12–15^ and others an increase in beta-band power^16–18^. Deep brain stimulation of the STN in PD patients has been shown to lead to a decrease in the power of spontaneous activity in the cortical sensorimotor beta and alpha (8–12 Hz) bands^19,20^ (but see also^16,21^).

Importantly, there is evidence that the beta-band changes are not in the same direction across the different stages of PD. For example, there are reports of increased cortical beta-band power in the early stages of PD^22^, whereas the later stages are associated with decreased beta-band power^23^. Further, the beta-band power is not the *only* feature of the sensorimotor rhythms that is altered in PD. Several studies have found a shift in the beta-band centre frequency (the frequency at which the power spectrum density peaks in the beta-band) towards a lower frequency in PD patients compared to healthy controls^24–26^. The shift towards lower beta-band centre frequency is more pronounced in PD patients with dementia^27–30^ and correlates with reduced cognitive ability^26,31^. Notably, the centre frequency shift is detectable already in the early stages of PD^25^ and does not seem to be affected by dopaminergic medication^32^. The changes in beta-band power and centre frequency in PD could indicate that different features of the oscillatory beta-band activity reflect different underlying neural functions expressed in the measured sensorimotor signals. Changes in beta-band power could be functionally related to sensorimotor disturbances, and changes in centre frequency could be related to cognitive function.

The characteristics of neuronal oscillatory activity may hold additional information on disease-related changes in PD^33^. Both beta-band power and centre frequency reflect a quantification of power spectral density (PSD). While these features can provide valuable information about disease-related changes in PD, the PSD quantification of a neural time series provides a static summary of the oscillatory activity across the entire time series. PSD does not account for inherent dynamics in this activity or changes in the time series on shorter time scales—as is prevalent in neural time series. The beta-band exhibits a great degree of variation over time and contains characteristic high-amplitude “bursts” that last about 50–200 ms, both in the cortical and sub-cortical beta-bands^34–37^. Functionally, the transient bursts appear to play a pivotal role in sensorimotor processing through the basal ganglia-thalamic-cortical network. For instance, the presence of a beta burst in the sensorimotor cortex close to a tactile stimulation decreased the likelihood of tactile detection^38^, and the rate of beta bursts is shown to decrease in the time leading up to a movement both in STN^39–41^ and in the sensorimotor cortex^42,43^.

In PD, quantification of beta-band burst activity from recordings in the STN has shown that beta-burst rate and duration are reduced by dopaminergic medication^44,45^ and deep brain stimulation^37^. Furthermore, PD patients exhibit a decrease in the rate of beta burst at the cortical level compared to healthy controls^14^. This decrease in beta burst rate is inversely related with increased severity of motor symptoms^46^; particularly bradykinesia and postural-kinetic tremor symptoms, but there is no evidence pointing to an effect of dopaminergic medication on cortical bursting properties^14^. Notably, the burst rate showed a higher sensitivity than PSD beta power for discriminating PD patients from healthy controls, demonstrating that the choice of method for analysing beta-band features influences the sensitivity of subsequent analyses. This is further complicated by the fact that in addition to disease-related changes, these features likely differ with age^43,47,48^, and the fact that most studies on oscillatory changes in PD come from studies with small sizes^49^. The central challenge is quantifying the measured neural signals to extract the disease’s relevant features from the signals, be it the spectral power, centre frequencies, or burst-like features.

**Fig. 1:**
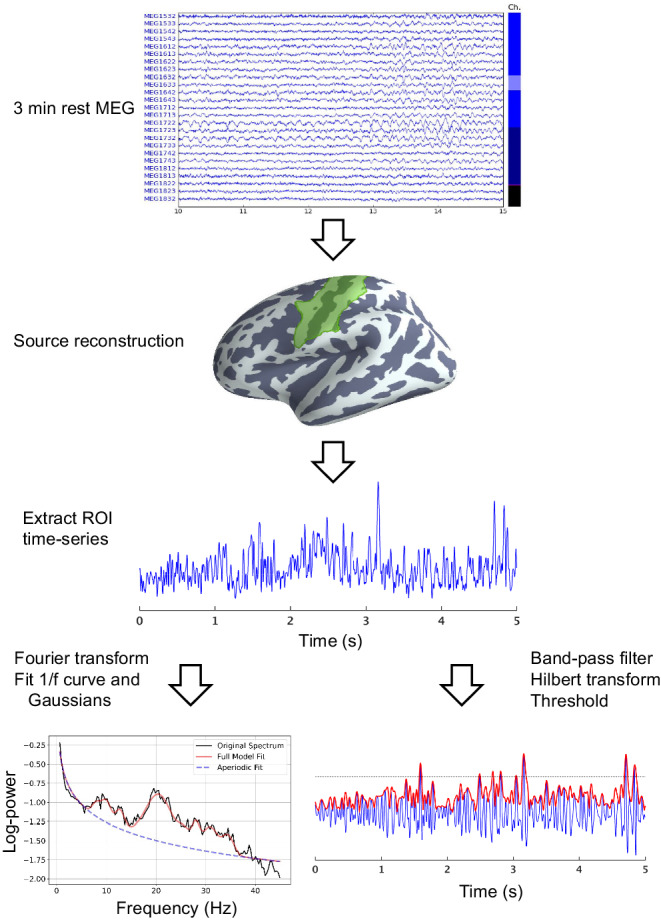
Overview of the data processing pipeline. Three minutes of resting-state MEG data was obtained from each participant. The signals were projected through a minimum-norm source reconstruction to extract the activity in the sensorimotor cortex. We did a frequency decomposition of the source reconstructed signal to calculate the PSD to which a 1/f and Gaussian curve were fitted to extract the PSD features (Table 2). In addition, we quantified sensorimotor bursts in the signal time series in the sensorimotor ROI by thresholding the envelope of the band-pass filtered (8–30 Hz) signal to the mu-beta frequency range.

In the current study, we aimed to explore which oscillatory- and non-oscillatory features of cortical sensorimotor activity differ between PD patients and healthy controls. As a secondary aim, we investigated the association of oscillatory- and non-oscillatory features with different motor symptoms in PD. We extracted the sensorimotor neural resting-state activity from source reconstructed resting-state MEG signals in the sensorimotor cortex (Fig. 1) and quantified the time-series in terms of the PSD in the canonical mu-band (8–30 Hz)^50,51^. In addition to the band-specific analysis, we compared the 1/f broadband characteristics of the PSD^52,53^, which previously has been shown to be altered in PD^14,54^. Finally, we compared features of the sensorimotor rhythm in terms of time-domain analysis of spontaneous transient bursts^14,38^. We tested the hypotheses of altered functional changes in PD by analysing how these features differed between PD patients and healthy controls and further investigated the interactions with age and sex. As ageing is associated with structural and functional changes in the sensorimotor cortex^55,56^, we investigated if the potential changes in sensorimotor activity in PD differed across age. Since both healthy ageing and PD disease progression are linked to thinning of the cortex^57,58^, we further included the thickness of the sensorimotor cortex in the analysis as a potential mediating factor on the sensorimotor activity that potentially also interacts with disease state.

The central question driving the exploratory analysis of the oscillatory- and non-oscillatory features was whether there would be differences between healthy controls and PD in features and if different features would be related to different functional changes. We hypothesised that individual oscillatory features would reflect different underlying neural functions in the sensorimotor system and thereby show different relationships to the clinical manifestations of specific motor symptoms in PD. We tested this hypothesis in two steps: first, in an exploratory analysis examining the inter-relationship between the different measures and group, age, sex, and cortical thickness. Second, by examining how the features explained the variation in severity of motor symptoms series in the sensorimotor ROI by thresholding the envelope of the band-pass filtered (8–30 Hz) signal to the mu-beta frequency range.

## Results

The study enroled 80 PD patients and 71 healthy controls balanced across gender and age, who all did three minutes of resting-state MEG. Table 1 presents a summary of the participants used in the analysis of oscillatory and non-oscillatory features in the cortical sensorimotor signals after data cleaning and pre-processing (see *Methods*).

**Table 1:** Group-level summary of the participants included in the analysis. Mean (standard deviation)

Measure	Parkinson’s patients	Healthy controls	Statistics
N	78	60	
Sex (female/male)	29/49	27/33	χ^2^ = 0.57, *p* = 0.45
Age	65.6 (9.5)	63.93 (8.4)	Welch’s t(138.0) = 1.08, *p* = 0.28
Disease duration	4.4 (3.7) years	–	–
LEDD	548 (273) mg	–	–
MDS-UPDRS-III	18.9 (10.8)	–	–
MoCA	26.1 (2.8)	26.2 (2.1)	Welch’s t(136) = 0.10, *p* = 0.92

For the first analysis, we investigated how features in the resting-state activity from the sensorimotor area quantified by features of the PSD and burst characteristics (see Table 2) differed as a function of the predictors: *group* (PD patients/healthy controls; Table 1), *age*, *sex*, *cortical thickness*, the two-way interactions between the predictors, and *age squared*.

**Table 2:** Summary explanations of the main outcome variables in the analysis

Variable category	Variable	Explanation
PSD	Beta power	The maximum peak in the 13–30 Hz band. Estimated as the height of the Gaussian function fitted to the PSD after regressing out the 1/f spectrum.
	Beta centre frequency (Hz)	The dominant frequency bin in the 13–30 Hz band. Estimated as the centre of the Gaussian function fitted to the 13–30 Hz range of the PSD after regressing out the 1/f spectrum.
	Alpha power	The maximum peak in the 8–12 Hz band. Estimated as the height of the Gaussian function fitted to the PSD after regressing out the 1/f spectrum.
	Alpha centre frequency (Hz)	The dominant frequency bin in the 8–12 Hz band. Estimated as the centre of the Gaussian function fitted to the 8–12 Hz range of the PSD after regressing out the 1/f spectrum.
	1/f offset	The intercept of the log-linear regression line estimated from the full PSD in the 0.5–40 Hz range.
	1/f exponent	The decay exponent (1/f^x^) of the PSD, corresponding to the slope of the log-linear regression line, estimated from the full PSD in the 0.5–40 Hz range.
Burst	Rate	The number of burst events in the time series divided by the length of the time series
	Duration (ms)	The time point from where the signal envelope rise above the threshold until the next time point it drops below the threshold.
	Interval (ms)	The time point from where the signal envelope drops below the threshold until the next time point it rises above the threshold.
	Amplitude	The maximum envelope amplitude within a burst event.

### PSD features

**Fig. 2:**
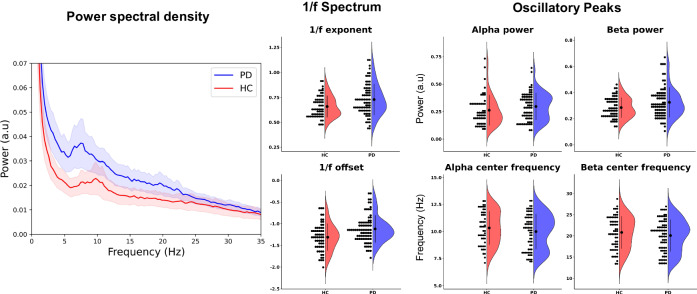
Summary of power spectral density (PSD) by group. Left: Grand average PSD (mean + standard error) for the PD group (blue) and healthy control group (red). *Middle*: the coefficient for the log-linear regression of the broadband PSD split by group. Right: the relative power and centre frequency of the oscillatory peaks in the alpha- (8–12 Hz) and beta bands (13–30 Hz) split by group after subtracting the fitted 1/f curve.

The PSD suggests an apparent group difference between PD patients and healthy controls in the alpha and beta bands (Fig. 2). However, analysing the oscillatory components of PSD by first adjusting for the broadband characteristic of the PSD^52^ removed the apparent group difference in the mu- and beta bands. Bayesian model comparison was used to test which predictors explained the variation in the PSD (Bayes Factors (BF) > 3 taken as the cut-off for substantial evidence for an effect of a given predictor^59^). The model comparisons instead showed evidence for group differences on the 1/f offset (BF = 55.7) and 1/f exponent (BF = 3.92). The 1/f offset was 24.0% [CI: 11.3:35.2%] higher for PD patients than healthy controls, and PD patients had 9.5% [CI: –1.7.4:22.2%] larger 1/f exponential compared to healthy controls, meaning that the PSD had a steeper decay for PD patients than healthy controls.

**Fig. 3:**
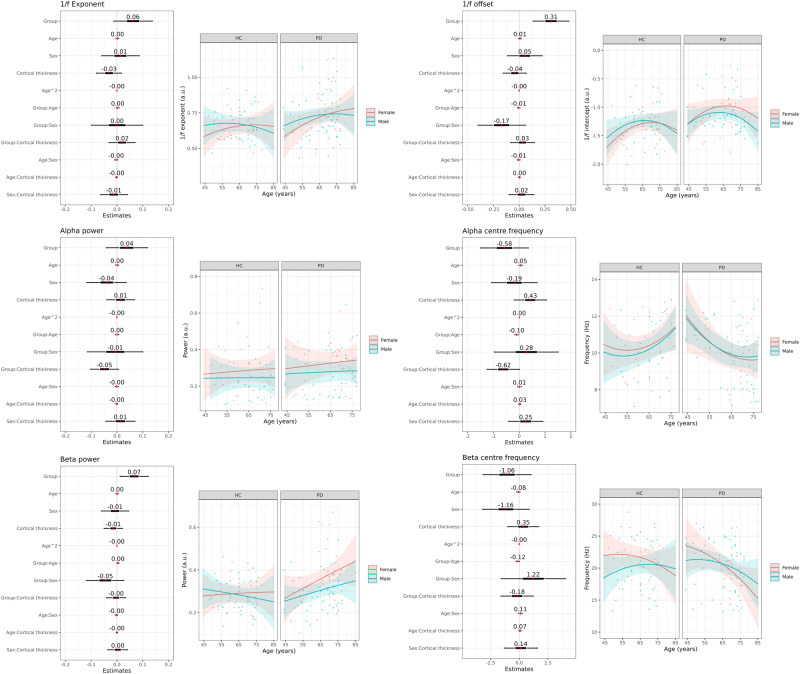
Regression analysis of PSD features. Standardised regression coefficients with 95%CI for the full regression models of 1/f exponent, 1/f offset, alpha power, alpha centre frequency, beta power, and beta centre frequency as a function of group, age, sex, cortical thickness, and the two-way interactions between these factors, together with model predictions across age span split between group and sex for each of the PSD features.

Analysis of the oscillatory peaks in the PSD after controlling for the 1/f characteristics did not yield evidence for an effect of group on either beta power (BF = 0.630) or alpha power (BP = 0.166). The beta- and alpha bands showed an interaction effect of Group and Sex on the beta centre frequency (BF = 5.58) and main effects of Group (BF = 3.06) and Sex (BF = 3.02). The alpha centre frequency showed an interaction between Group and Cortical Thickness (BF = 6.50). There was no evidence of an effect beyond the threshold for substantial evidence on any other PSD features. The coefficients and model predictions of the full models for all outcome measures are presented in Fig. 3.

Given the explorative nature of the analyses, it is of interest to mention that there was anecdotal evidence (BF > 1.5) of an interaction effect of Group and cortical thickness (BF = 1.92) and a main effect of Sex (BF = 1.87) on 1/f exponent, interaction effects of Group and cortical thickness (BP = 1.92) and Sex and Cortical Thickness (BF = 1.87) and a main effect of Cortical Thickness (BF = 1.76) on beta centre frequency, and main effects of Cortical Thickness (BF = 2.00) and interaction between Sex and Group on alpha centre frequency (BF = 2.57).

To explore how the choice of not correcting the PSD by removing the 1/f characteristic before quantifying oscillatory power in the alpha- and beta frequency bands affected the analysis of the PSD, we repeated the analysis of power and centre frequency for the alpha- and beta band by quantifying alpha- and beta power and centre frequency as the peak within the respective bands. This analysis yielded no evidence for band power differences (all BF < 1). For the uncorrected PSD, the beta centre frequency showed interaction effects between Group and Sex (BF = 3.46) and Group and Cortical Thickness (BF = 4.34) and main effects of Sex (BF = 6.86) and Group (BF = 10.95). In line with the analysis after removing the 1/f characteristic from the PSD, the PD patients had a lower beta centre frequency, with males being lower than females and male patients being even lower than female patients. The alpha centre frequency showed an interaction effect between Group and Cortical Thickness (BF = 6.52), showing a slightly stronger negative association between alpha centre frequency and cortical thickness.

### Burst features

**Fig. 4:**
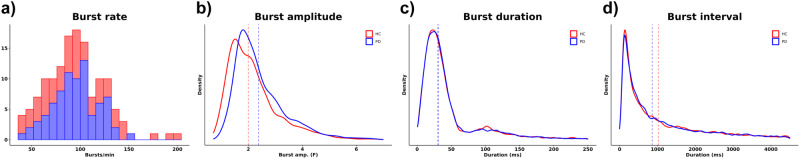
Group-level summary of burst features. Split between the PD (blue) and healthy controls (red). **a** Histograms of burst rate count. **b** Probability density of burst amplitudes pooled within group. **c** Probability density of burst duration pooled within group. **d** Probability density of burst interval pooled within group. Horizontal dashed lines indicate the group-level means.

The view of sensorimotor oscillatory activity has recently changed from analysing such activity as a steadily oscillating signal to a view where activity is considered to be composed of transient oscillatory bursts. We compared features of the sensorimotor rhythm in terms of time-domain analysis of such transient bursts. Group-level summaries of the burst features are presented in Fig. 4.

Model comparison to test which predictors explained the variation in the burst features gave evidence for an interaction effect between group and age on the burst rate (BF = 3.34) and a main effect of sex on the burst rate (BF = 3.80). The age-related effects from the model amount to a fall in burst rate of –0.6% per year [CI: –1.3:0.1%] for PD patients, whereas for healthy controls, the model predicted a stable burst rate across age with a change of 0.5% [CI: –0.2:1.1%] per year.

**Fig. 5:**
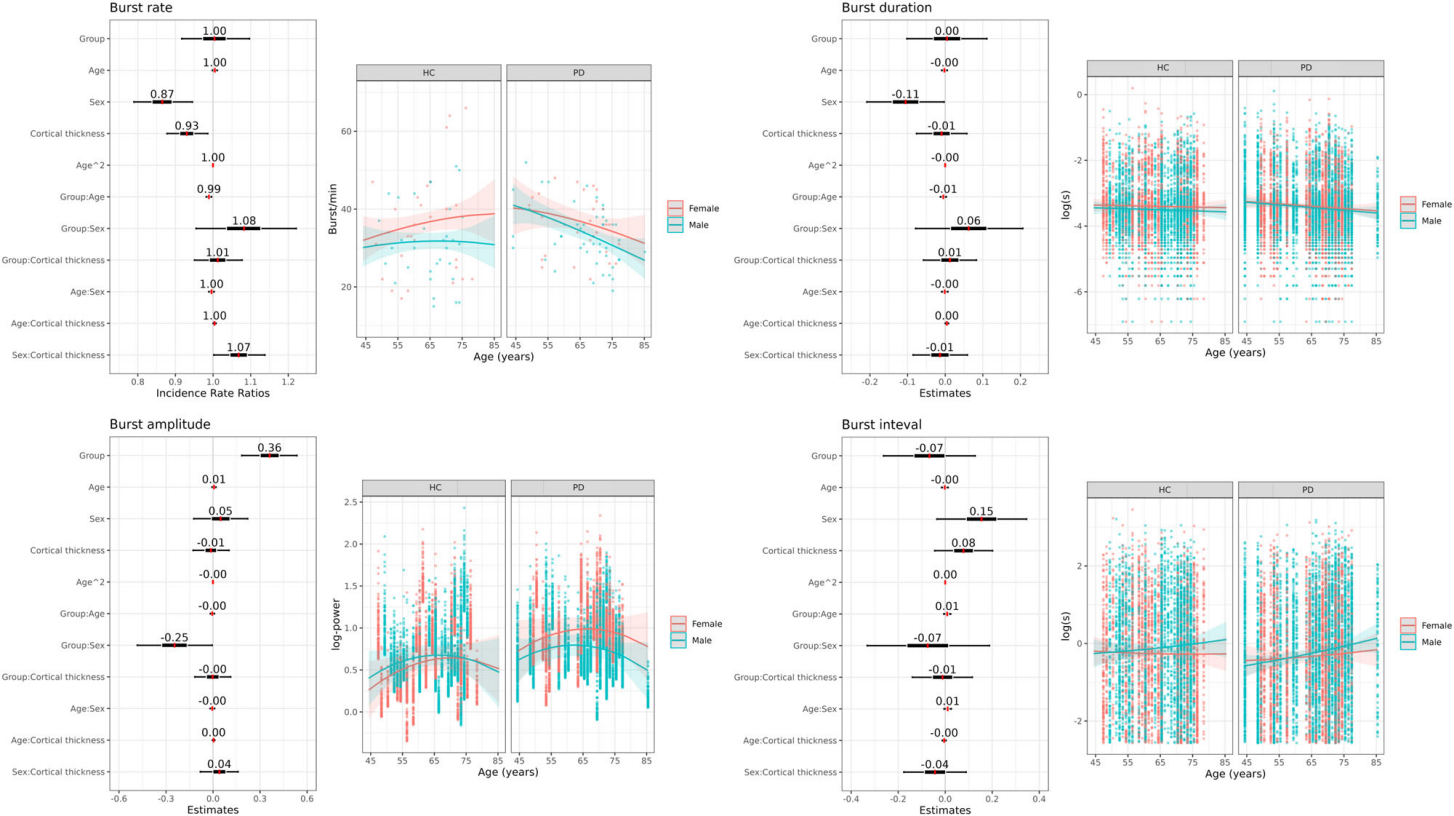
Regression analysis of burst features. Standardised regression coefficients with 95%CI for the full regression models of burst rate, duration, interval amplitude as a function of group, age, sex, cortical thickness, and the two-way interactions between these factors, together with model predictions across age span split between group and sex for each of the burst features.

The burst amplitude showed an interaction effect of Group and Sex (BF = 3.22) and a main effect of Group (BF = 32.3). The analyses did not reveal any effects of Group, Age, Sex, or Cortical Thickness on the burst duration or interval. All model coefficients for the burst features are displayed in Fig. 5.

### Clinical symptoms and oscillatory features

**Fig. 6:**
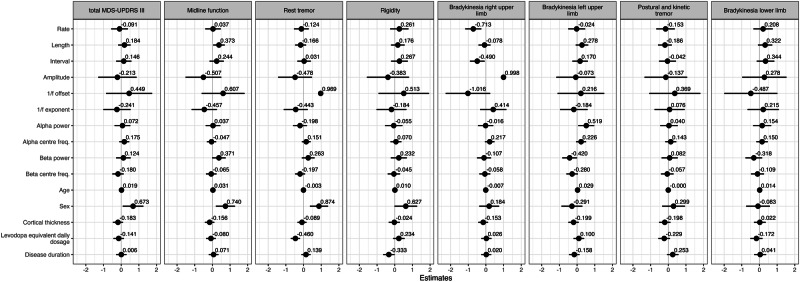
Regression analysis of motor symptoms. Standardised regression coefficients with 95%CI for the regression analyses of sensorimotor signal features on clinical motor symptom ratings in PD.

In the second analysis, we tested for associations between motor symptoms and the features of the sensorimotor signal in the PD group. All sensorimotor signal features listed in Table 2 were used as predictors in a multiple regression analysis that further included age, sex, disease duration, levodopa equivalent daily dose (LEDD), and cortical thickness. The standardised regression coefficients of each predictor variable on the motor symptoms measured with MDS-UPDRS-III^60^ are presented in Fig. 6.

Model comparison of multiple regression models showed evidence that burst rate (BF = 48.48) and burst interval (BF = 4.23) were negatively associated with upper limb bradykinesia on the contralateral side. The negative direction means that reduced burst rate was associated with increased severity of bradykinesia. Furthermore, there was substantial evidence that burst duration was positively associated with the total MDS-UPDRS III score (BF = 10.61) and with axial symptoms (BF = 3.56). That means that longer bursts were associated with higher symptom burden. The analysis yielded no evidence for effects of other sensorimotor signal features on symptom ratings for rest tremor, rigidity, rest tremor, postural/kinetic tremor, or lower limb bradykinesia.

The analysis yielded substantial evidence for a difference between male and female patients on total MDS-UPDRS III scores (BF = 3.79), axial symptoms (BF = 13.32) and rest tremor (BF = 132.63), and for an effect of LEDD on rest tremor (BF = 198.68). There was anecdotal evidence for an effect of age on axial symptoms (BF = 2.99), sex (BF = 1.52), and disease duration (BF = 2.03) on rigidity, as well as anecdotal evidence for an effect of alpha centre frequency on ipsilateral upper limb bradykinesia (BF = 2.68).

## Discussion

This study aimed to explore how different features of cortical somatosensory oscillatory activity at rest differed between PD patients and healthy controls across age and sex—and how these features relate to motor symptoms in PD. The analysis of spontaneous sensorimotor bursts showed evidence of an increased age-related reduction in PD patients compared to healthy controls. Notably, our current results show that the reduced burst rate in PD is not a static group-level difference but interacts with age, such that a steeper age-related reduction in burst rate is seen in PD compared to healthy controls.

In the analysis of the time series PSD, the PD group showed a higher broadband 1/f offset compared to healthy controls and a steeper 1/f decay (i.e., larger exponent) of the PSD than the healthy controls. The PSD analysis did not yield evidence for differences between patients and healthy controls in oscillatory power.

We further explored whether different oscillatory features reflect distinct underlying functional neural properties and manifest as different motor symptoms in PD. The results showed that a reduced burst rate and the interval between bursts were accompanied by increased bradykinesia severity in the contralateral side of the body in PD, confirming previous findings from our group^14^. On the other hand, the analysis showed that the burst duration was associated with axial symptoms in PD and the overall degree of motor dysfunction. This relationship was exclusive for burst, as we observed no statistically evident relationships between PSD features and clinical motor symptoms.

The cortical sensorimotor activity of the PD patients differed from that of healthy controls. However, we did not find evidence that the sensorimotor activity differed when comparing the PSD in the canonical sensorimotor mu and beta bands, independent of whether the bands were analysed as the raw PSD containing the 1/f characteristic of neural time series or if the 1/f characteristic was first removed before quantifying the oscillator peaks. The notable difference between groups in the PSD was the difference in the 1/f broadband characteristics. The evidence of a group difference in the spectral broadband 1/f characteristics of the signal adds to the growing evidence that a focus on neural activity as narrow-band steady oscillations—e.g. narrowly focusing only on the beta-band power—could potentially miss essential aspects of the neural signals for understanding mechanistic changes in PD^54,61^.

Theoretically, unaccounted-for systematic differences in either PSD offset or decay exponent could lead to a false conclusion that there is a difference in the oscillatory response^52,53^. In the current study, we repeated the analysis of the oscillatory features (i.e., alpha- and beta power and centre frequency) to give a picture of how correcting the PSD for the 1/f characteristic affected the conclusions. Surprisingly, the analyses with and without the 1/f characteristic removed yielded converging findings: neither analysis provided evidence of power differences in the alpha- and beta bands but gave evidence that the beta centre frequency differed between groups and sex, and the alpha centre frequency entailed an interaction between group and cortical thickness. The main conclusion here is that even if the narrow-band analyses yielded similar results, the broadband 1/f characteristic of the PSD provides further relevant information that distinguishes PD patients from healthy controls in addition to the narrow-band oscillations. Widening the quantitative analysis of PSD rather than focusing exclusively on narrow-band power is of potential clinical value.

While there is converging evidence that changes in the 1/f characteristic are important for understanding neural functions, it is still not understood what the functional relevance is. A possible explanation is that the steepness of the spectrum, i.e., the 1/f exponent, is an expression of the relative excitation-inhibition balance in the source of the electrophysiological signal. Simulating the electric fields from the combination of the relatively slow inhibitory synaptic (GABAergic) transients and the relatively faster excitatory (glutamatergic) currents shows that varying the relative excitation-inhibition balance leads to changes in the 1/f exponent^62^. The difference in 1/f characteristic between PD patients and healthy controls might reflect either increased inhibitory GABAergic signals or, conversely, a reduction in glutamatergic currents in the sensorimotor cortex. This would align with a metanalysis of fMRI studies showing a general reduction of activity in the motor cortex in PD^63^. However, it is also possible that the 1/f characteristic is not only driven by local changes but could be an expression of slow dynamics of the ascending inputs to the cortex, thus leading to higher power in lower frequencies. At the current stage, there is a need to further bridge how 1/f features in the signals are linked to disease mechanisms.

Despite several studies providing evidence that the 1/f characteristic of the PSD is altered in PD, none of these studies have found that the clinical manifestation of motor symptoms was related to the 1/f characteristic^14,54,61^. Here, in a much larger sample, we still do not see evidence that the 1/f offset and exponent are directly associated with motor symptoms (as assessed by the UPDRS-III). This points to the 1/f features being independent of the dopaminergic system and reflecting either local excitation-inhibition balance or ascending slow drive from the thalamus. On the other hand, the motor symptoms in PD depend on the dopaminergic networks and clearly improve with dopaminergic medication. This could also explain why the 1/f exponents have previously been found not to differ depending on dopaminergic medication in PD^14,54,61^, and the increase in 1/f exponent in PD compared to healthy controls is seen through most of the cortex, excluding the frontal cortices rather than just the areas that receive input from the dopaminergic areas^54^. Though the present study focused on the activity of the sensorimotor cortex and the motor symptoms, inspection of the correlation between all signal features and clinical test scores in the current study showed a significant negative correlation between 1/f exponent and cognitive assessment with MoCA (supplementary material), which could hint at a relation between increased 1/f slope and cognitive decline. Indeed, a recent study found that changes in the aperiodic 1/f in the frontal cortex are predictive of cognitive decline in PD^64^ and another showed a larger 1/f exponent in patients with Lewy body dementia compared to PD patients without dementia and healthy controls^65^. The change in 1/f characteristic of the cortical activity appears relevant to understanding cognitive symptoms in PD.

Analysis of neural time series by frequency decomposition is a powerful tool to extract and summarise signal features, but the method has limitations in what one can infer. In the time domain, increased oscillatory power can reflect both increased burst duration and change in burst amplitude and an expression of true sustained oscillations in the signal^36,66^. The neural time-series can express both a degree of sustained oscillations while also exhibiting variation in the degree of transient bursts—e.g., a signal of steady oscillation with transient high-amplitude bursts. The presence of sustained oscillations in the sensorimotor rhythm might reflect a higher level of functional inhibition of sensorimotor information; as is seen in recordings from STN^5^ and, to some extent, also at the cortical level^38,67^. However, sustained oscillations are not in contrast to the bursting properties of the sensorimotor rhythm. The various features of the bursts are likely expressions of distinct underlying pathophysiological processes. We found evidence that burst rate and amplitude varied depending on group and were inversely related to bradykinesia. We further found that the longer burst durations—after regressing out the contribution of other features—were associated with increased axial symptoms.

Bursting properties of the cortical sensorimotor neural activity are proposed to occur due to long-range input through the ascending thalamic-cortical connection to the cortex, leading to an increase in the local neural excitation and resulting in a burst of synchronous activity^36^. The observed disease-related changes in spontaneous cortical bursts, in the form of a more rapid decrease in burst rate over age for PD patients, could reflect inhibition of these projections along the thalamic-cortical pathways caused by disturbances in the dopamine-dependent structures projecting to the cortex. The association of beta burst duration with midline functions is in line with previous studies reporting that longer bursts in the subthalamic nucleus are associated with the postural-instability and gait disorder (PIGD) motor phenotype of PD^68,69^, Freezing of Gait and other gait features^70^. However, we did not find significant group differences in burst duration in the current study—in line with previously reported findings on cortical bursts in PD^14^—supporting the view that the central mechanisms of the cortical bursts are not primarily affected in PD—instead, it is the *rate* of bursts that is reduced at the cortical level.

The sub-cortical beta-band activity is influenced by the activity of dopamine-responding neurons^5,6,45^. The effect of dopamine and dopaminergic medication on the cortical beta-band is likely mediated by the dopaminergic neurons projecting to the cortex that terminate in the pre-frontal cortex but also, to a lesser extent, in the primary sensorimotor cortex^71^. The differences in the cortical sensorimotor burst rate in PD might be an indirect effect of the loss of dopamine and changes in the beta band in the sub-cortical structures projecting to the sensorimotor cortex. The notion that the cortical sensorimotor activity is only indirectly related to dopamine depletion in PD is further supported by findings from animal studies showing that 6-hydroxydopamine injections lead to exaggerated beta-band oscillations only after several days had passed, suggesting that oscillatory changes occurred as an indirect compensatory effect after dopamine depletion rather than a direct consequence of the depletion itself^7^. The indirect influence of dopamine on the cortical beta-band might also explain the often weak or even absent effect of dopaminergic medication on cortical beta-band activity^12–15^.

The current study cannot directly address the role of dopamine on cortical oscillations since all patients in the study were tested on medication. However, a recent study found that cortical burst characteristics measured with MEG were influenced by DBS therapy in PD and normalised the bursting characteristics during DBS to resemble the burst characteristics of healthy controls^46^. This further supports that cortical bursting activity is mediated by subthalamic projections.

We explored how age-related differences in cortical sensorimotor neural activity might interact with disease-related changes in PD. Age-related effects on spontaneous sensorimotor activity are commonly dealt with by matching the age distributions of the patient group and the healthy control group—usually within a narrow age span. The analysis showed age-related differences in burst rate, with PD patients showing a more considerable reduction of bursts as a function of age than healthy controls. We found no evidence of change in burst rate over age in healthy controls, which is in line with previous studies on elderly healthy controls^47^—though another study has found an age-related decrease in burst rate from young adulthood to old age in healthy participants^43^. The main finding in the present study is that the burst rate decreases for PD patients across the age span we investigated. The reduction in burst rate with age in PD seems in accordance with the fact that higher age at PD onset is associated with a faster disease progression and more rapid decline in motor function^72^, though a longitudinal design is needed to confirm the relation between disease progression, reduction in burst rate, and age.

A previous study reported that old age was associated with longer burst duration when comparing healthy young and elderly participants^48^. However, we did not find evidence of any age-related effects on burst duration when looking across age rather than comparing young- and elderly adults. However, our study did not include a group of young participants, so the change in burst duration likely occurs from young adults to adulthood rather than in late adulthood. Similarly, another study looking at burst features across age with age as a continuous variable did not find an association between age and burst duration—only burst rate^43^.

We included cortical thickness measures within the same ROI from which we extracted the functional time-series, as we hypothesised that age-related effects upon the functional measures might be mediated through the age-related structural changes in the cortex. However, despite the negative correlation between age and cortical thickness (see supplementary material), we did not find substantial evidence that cortical thickness affected burst features. In all our analyses, we only found evidence that cortical thickness was associated with the oscillatory centre frequency in the alpha band in and interaction with group. An explanation might be that such slowing is more pronounced in PD patients with dementia^27–30^ and correlates with cognitive ability^26^. The PD patients in the current study did not differ in their cognitive ability from the healthy controls. Furthermore, we focused on the activity in the sensorimotor cortex, whereas the slowing of alpha and beta PSD is usually found in frontal areas and globally throughout the brain^25,28,31^.

We included sex to explore if disease-related changes in sensorimotor oscillatory activity differed between males and females, as there are well-documented sex differences in the manifestation of PD^1,73^. Male sex is a risk factor for developing PD, with an average incidence ratio of approximately 2:1 male-female ratio across all stages of the disease^74^. The disease onset is, on average, two years earlier in males than females and differs in the initial manifestation of symptoms, with women more likely to develop tremor-related symptoms and men more likely to develop rigidity^75^. In line with that, the regression analysis of motor symptoms showed evidence for a difference in overall motor symptoms, midline function and rest tremor between male and female patients.

The analysis of the sensorimotor signal features showed evidence of differences between males and females in the burst rate. The sex difference in burst rate did not depend on whether it was healthy control or PD patients. The burst amplitude, on the other hand, showed an interaction between group and sex. In line with our finding, a previous study investigated sex differences in PD in recordings from STN and found that females had higher alpha- and beta power than males—however, only in the OFF state, whereas females only had higher power in the high gamma band when ON medication^76^. A possible factor behind the sex differences in PD is the contribution of sex hormones on the nigrostriatal pathway linked to the deterioration of the dopaminergic system, where testosterone levels appear to enhance dopamine loss, while oestrogen has been identified as a neuroprotective agent for PD. Oestrogen has been demonstrated to influence incidence levels of PD, while menopause-related variations in oestrogen levels are linked to variations in PD symptom severity^77^. At the current stage, it is unclear if oestrogen sex hormones influence oscillatory bursts in the STN and the sensorimotor cortex. These findings illustrate the need for further studies into sex-specific changes in neural function and how they manifest and relate to PD.

The present study quantified the neural time series from the sensorimotor cortex based on pre-defined summary measures of its PSD and burst properties. A limitation of our study for understanding the extent of changes in oscillatory sensorimotor activity is the focus on different features within a narrow ROI, which ignores other types of measurements that are potentially relevant to understanding the development of PD and motor symptoms: for example, the long-range connectivity between the sensorimotor cortex and other cortical areas and the connections between the sensorimotor cortex and the basal ganglia and thalamus. Treating the activity in the sensorimotor cortex as a single time series also means that we remove the sensitivity to spatial features of the signals, e.g., focal versus spatially blurred activity in one group or the other. If the oscillatory activity extends over a larger cortical surface area, that signal will also manifest as power differences in the measured signal^66^. There are potentially other features to be uncovered, and future studies may explore how the PSD- and burst features further interact with other aspects of brain activity in the global function of the brain to fully understand the interaction between functional and structural changes in PD.

We investigated a relatively large cohort of PD patients and healthy controls (for a neuroimaging study) to make meaningful inferences about how age and sex interact with the group-level difference between PD patients and healthy controls; however, a limitation is that our study is cross-sectional. Therefore, the age-related effects we report here are limited by what is possible from cross-sectional inference. The variation from individual to individual in the various measurements may obscure fine-grained effects. Ideally, we will follow this cohort longitudinally to estimate the development trajectories of the sensorimotor oscillatory activity in PD and in healthy ageing.

For understanding the functional changes in the sensorimotor cortex in PD, the most limiting factor for our study is that we only acquired MEG from PD patients while in the ON medicated state. This means that the PD measurements do not reflect the worst manifestation of the disease when dopamine is depleted. This would have represented a state with more severe motor symptoms and thus a truer state of the disease to uncover the disease mechanisms. This could have led to larger group differences and potential group effects we did not find evidence for, as the medication normalises the activity in the PD patients to be more like the healthy controls. The normalising effect of dopaminergic medication on neural oscillations is, for example, well documented in deep brain recordings^5,9,11,45^. However, the effect of dopaminergic medication is not as apparent on neural signals measured at the cortical level. The motivation for only recording MEG for PD patients in the ON state was that the features we wanted to investigate—i.e., burst and 1/f features—were not previously shown to differ depending on medication in PD^14,54,61^. We acknowledge that with the sample size we achieved in the present study, we might have been able to detect effects that were otherwise not statistically detectable in the previous smaller study, not to mention that it would have been possible to provide an answer to how the medication would have affected the signal features. However, in designing the study, we prioritised achieving a large sample of participants over doing repeated measurements on and off medication. Future studies should investigate whether the effects we report here are indeed affected by Parkinsonian medication.

Sensorimotor activity measured non-invasively with MEG/EEG contains rich information about the functional state of the sensorimotor system and how it changes in PD. The central challenge is quantifying the measured neural signals to extract the disease’s relevant features from the signals, be it the spectral power, centre frequencies, or burst-like features. Finding features of neural signals that can explain disease mechanisms or symptoms, even if extracted along with a reduced number of dimensions, will be helpful if they provide adequate information about the disease- or symptom-state. Further characterisation of the association between features in the non-invasive brain signals and motor symptoms can potentially be a valuable tool to aid in diagnosis and treatment evaluation. Understanding how features in the neural time series are related to motor symptoms in PD will also help develop non-invasive neural stimulation that can potentially relieve motor symptoms^37,78^.

## Methods

### Participants

Eighty PD patients (age 44–85; 32 female) and 71 healthy controls (age 46–78; 46 female) participated in the study. The study was approved by the regional ethics committee (Etikprövningsnämden Stockholm, DNR 2019-00542) and followed the Declaration of Helsinki. All participants gave written informed consent before participation.

The PD patients were recruited from the Parkinson’s Outpatient Clinic, Department of Neurology, Karolinska University Hospital, Stockholm, Sweden. The healthy controls were recruited by advertising or amongst spouses of PD patients. 22 participants (18 patients, 4 healthy controls) were included from a previous study^14^ who were qualified based on the recruitment criteria of the present study and had done the same MEG and MRI procedures as in the present study. All data were reanalysed following the procedure described below.

The inclusion criteria for the PD group were a diagnosis of PD according to the United Kingdom Parkinson’s Disease Society Brain Bank Diagnostic Criteria with Hoehn and Yahr stage 1–3^79^. Inclusion criteria for the control group were not having a diagnosis of PD, no form of movement disorder, and no history of neurological disorders, epilepsy, or psychiatric disorders.

Exclusion criteria for both groups were a diagnosis of major depression, dementia, history or presence of schizophrenia, bipolar disorder, epilepsy, or history of alcoholism or drug addiction according to the *Diagnostic and Statistical Manual of Mental Disorders*^80^.

One participant declined to do the MRI scanning, one participant had a scanner malfunction during MRI acquisition, and 11 participants had their MRI scans cancelled due to the COVID-19 pandemic and were not included in the analysis. In total, two PD patients and 11 healthy controls were excluded from the analysis. Table 1 is a summary of the participants included in the analysis.

The PD patients participated in the study while on their regular prescribed dose of medication. Levodopa equivalent daily dose was calculated according to Tomlinson et al. ^81^. Motor symptoms in the PD group were assessed using the motor section of the Movement-Disorder Society Unified Parkinson’s Disease Rating Scale (MDS-UPDRS-III)^60^. Global cognition was assessed with the Montreal Cognitive Assessment battery (MoCA)^82^.

### MEG recordings

MEG data were recorded with a Neuromag TRIUX 306-channel MEG system, with 102 magnetometers and 102 pairs of planar gradiometers. Data were sampled at 1000 Hz with an online 0.1 Hz high-pass filter and 330 Hz low-pass filter. The MEG scanner was located inside a three-layer magnetically shielded room (Ak3B, Vacuumschmelze GmbH) with internal active shielding active to suppress electromagnetic artefacts. The subjects’ head position and head movements inside the MEG scanner were measured during recordings with head-position indicator coils (HPI) attached to subjects’ heads. The HPI location and additional points sampled uniformly across the subjects’ head shape were digitalised with a Polhemus Fastrak motion tracker before the measurements. Horizontal and vertical electrooculogram (EOG) and electrocardiogram (ECG) were recorded simultaneously with the MEG.

We recorded three minutes of resting-state MEG while the participants sat with their eyes closed. The participants were instructed to close their eyes and relax. The recordings began after assuring the participant sat still with their eyes closed.

The participant’s head movements during the recording were measured by continuously sampling the HPI location. The average head movements were not significantly different between groups (t(115.8) = 0.55, *p* = 0.58).

### MRI acquisition

3D T1-weighted magnetisation-prepared rapid gradient-echo (MPRAGE) sequence structural images (voxel size: 1x1x1 mm) were obtained on a GE Discovery 3.0 T MR scanner for morphological analysis and creating source spaces for MEG source reconstruction. Multi-echo “FLASH“^83^ images were obtained to create volumetric headmodels for MEG source reconstruction (see below).

### MRI processing

The MRI images were processed with Freesurfer^84^ (v. 5.3) to get surface reconstructions of the cortical mantle. The surfaces were obtained with the automatic routine for extracting cortical surfaces in Freesurfer from the individual T1-weighted MRI.

We defined the cortical sensorimotor area by segmenting the cortical surface using the anatomical labels provided by Freesurfer automatic labelling^85^. The analysis focused on a region of interest (ROI) consisting of the left pre- and post-central gyri and central sulcus. The pre/postcentral gyri were combined because a biomagnetic source on either sulci wall will leave a trance on the other side due to the close distance and the field spread of MEG signals. The ROI was defined for each subject based on the individual cortical reconstructions. The average cortical thickness in the ROI was estimated with Freesurfer^86^.

### MEG pre-processing

The MEG data was processed by applying temporal signal space separation (tSSS) to suppress artefacts from outside the scanner helmet and correct head movement during the recording^87^. The tSSS had a buffer length of 10 s and a cut-off correlation coefficient of 0.95. Movement correction was done by shifting the head position to a position based on the median of the continuous head position during the three-minute recording.

The MEG data processing and source reconstruction was done with MNE-Python^88^ in Python 3.8. First, we marked data segments containing muscle artefacts and SQUID jumps with the automatic artefact detection in MNE-Python. The data was filtered with a 48 Hz low-pass zero-phase FIR filter with a 12 Hz transition bandwidth and 50 Hz notch filter to remove line noise. The continuous data were cut into 1.0 s epochs, and epochs with muscle artefacts or extreme values (5000 fT for magnetometers and 4000 fT/cm for gradiometers) were rejected. Between 0 and 65% (median: 6.0%) of data was rejected, resulting in 63.0–180 s (median: 174.0 s) of useful MEG data per participant. The remaining data length was not significantly different between groups (Wilcoxon rank sum test, *p* = 0.98). We then performed an independent component analysis (ICA) using the *fastica* algorithm^89^ to identify artefacts from blinks and heartbeats. Components showing correlation with the EOG and ECG were removed from the raw data. Between 0–5 (median 3) components were removed per participant. The number of removed ICA components was not significantly different between groups (Wilcoxon rank sum test, p = 0.71).

We then applied source reconstruction using noise-weighted minimum-norm estimates^90^. The noise covariance matrix was estimated from two minutes of empty room MEG data recorded before each session. The source space consisted of 5124 evenly spaced points sampled across the white matter surfaces. The inner skull boundary was estimated from the multi-echo MRI to create a single shell volume conductor model. The time series from the sensorimotor ROI (see Fig. 1) was extracted from the estimated source time series by singular value decomposition of all source points within the ROI. The resulting time series were used to calculate the PSD and oscillatory bursts.

### Power spectral analysis

We analysed the spectral properties of the sensorimotor activity by calculating the PSD from 0.5 to 40 Hz across the entire cleaned ROI time series using Welch’s method by segmenting the continuous data into 3.072 s segments with 50% overlap and averaging the PSD across the segments.

Since the narrow-band beta power in the PSD is dependent on the broader features of the broadband spectrum, we further analysed the 1/f broadband characteristic of the sensorimotor activity as this could play a role in the functional properties of the beta-band and has been shown to differ between healthy control and PD patients^14^. We used the *fitting oscillations & one over f* (FOOOF) toolbox^52^ to analyse the 1/f broadband characteristic of the PSD (offset and exponent) and the oscillatory peaks in the canonically defined beta band (13–30 Hz) and alpha band (8–12 Hz). A log-linear regression is fitted to the PSD and subtracted before fitting Gaussian functions to the peaks in the PSD. The midpoint of the Gaussian function fitted to a given frequency band corresponds to the peak frequency in that frequency band, and the height represents the signal power. A new log-linear function is fitted to the PSD after subtracting the Gaussian function to estimate the 1/f characteristic. The parameters of the fitting procedure were set to a maximum of 8 peaks, peak threshold of 2, and a minimum peak height of 0.05, with a bandwidth between 0.75 and 12 Hz. The goodness-of-fit for the model fit was at a median R-squared of 0.968 across all subjects (range 0.89-0.99) and did not differ between groups (Welch-t(133.41) = –1.11, *p* = 0.269). All participants showed a discernible beta peak in the PSD. Nine PD patients and nine healthy controls did not show a peak in the PSD alpha band (no difference between groups, χ^2^(1) = 0.12; *p* = 0.73). The participants who did not show an alpha peak were not included in the analysis of the alpha band.

We repeated the analysis of alpha- and beta power and centre frequency taken directly from the PSD without doing the FOOOF procedure. Power and centre frequency were found by finding the peak in the uncorrected PSD for the alpha- and beta-frequency range, respectively.

### Burst analysis

To calculate the burst properties of the sensorimotor activity in the time domain, we band-pass filtered the time-series with an 8–30 Hz band-pass filter using FieldTrip^91^ in MATLAB (R2016b; MathWorks Inc.) and calculated the Hilbert envelope of the signal. The burst threshold was defined as two times the median of the signal. The burst onset was defined as the time-point where the signal first reached half the max amplitude of the burst and ended at the time-point where the signal again dropped below half the max amplitude of the burst. The *burst amplitude* was defined as the maximum value of the burst. The burst *duration* was defined as the time from burst onset to burst end. The *burst interval* was defined as the time from the end of a burst to the time-point where the next burst began.

### Statistics

*Analysis of sensorimotor rhythm features*. The main analyses tested the effect of *group* (PD patients/healthy controls), *age*, *sex*, and *ROI cortical thickness* on the features listed in Table 2. For the PSD features, we modelled the outcomes as a linear function of group (PD patients/healthy controls), age, age squared, sex, and cortical thickness with linear regression in *R* (v. 4.1.3)^92^. The regression models were fitted to the data for each participant with all factors and up to their two-way interactions between the four predictors. Gaussian regression models were estimated for each feature, except for the burst rate (burst per minute), which was modelled with Poisson regression for count data using the same predictor variables. Before fitting the regression models, burst duration, burst interval, and burst amplitude were log-transformed.

Hypothesis testing was done by computing the Bayes factor (BF) for the model with a given predictor (H1) versus the model without the predictor (H0). Given that the study is an explorative analysis, Bayesian is the appropriate statistical approach as the BF tells how much more likely the observed data is under the alternative model (H1) versus the null model (H0) and avoids the multiple comparison problem of frequentist hypothesis testing. The model comparison approach furthermore circumvents issues with interpreting *p* values of individual regression coefficients due to internal correlation between predictor variables. Following the conventional interpretation of BFs, we used BF > 3 as a cut-off between anecdotal evidence and substantial effects^59^. The model comparison was done by sequentially adding predictors and comparing the variance explained between the model with the predictor (H1) and the previous model without the predictor (H0). The BFs for all comparisons, model coefficients and *P*-values for model coefficients are presented in the supplementary material.

*Clinical scores and sensorimotor oscillatory features*. The MDS-UPDRS-III scores were divided into subscales based on symptoms: *midline function*, *rest tremor*, *rigidity*, *upper-body bradykinesia*, *postural and kinetic tremor*, and *lower limb bradykinesia*; according to Goetz et al. ^93^. Each of the symptom composite scores were analysed by multiple regression; modelled as a function of the burst rate, median burst duration, median bursts interval, median burst amplitude, PSD 1/f offset, PSD 1/f exponent, PSD beta power, PSD beta centre frequency, PSD alpha power, and PSD alpha centre frequency for each PD patient. The models further included the age, sex, disease duration, LEDD, and cortical thickness to regress out the contribution hereof and estimate the relative effect size of each signal feature. All symptom ratings and continuous predictor variables, except age, were z-transformed to get the standardised effect size. Significance testing was done by removing one predictor from the model and calculating the BF between the full model (H1) and the model without the predictor (H0) using the BIC approximation for BFs. The BFs for all comparisons are presented in the supplementary Tables.

### Reporting summary

Further information on research design is available in the Nature Research Reporting Summary linked to this article.

### Supplementary information

Supplementary material

Reporting Summary

## Data Availability

The full dataset cannot be made publicly available, as the ethical permits for the study do not allow for open data sharing. We have released parts of the MEG data used in this analysis in anonymised form^94^. MRI data cannot be shared, but individually modified template MRIs to recreate the MEG source reconstruction^95^ are part of the released dataset. The data is available through the EBRAINS data-sharing platform^96^. Access to the data requires that the acceptance of the EBRAINS Data Usage Agreement for human data requires all users to create an account and identify themselves using an institutional email address. For a detailed description of the data, we refer to the accompanying data descriptor^94^. The scripts used to process the data and run the analysis presented in the paper are available at: https://github.com/natmegsweden/PD_beta_bursts2.
