# Supplementary material for: Oscillatory and non-oscillatory features of the magnetoencephalic sensorimotor rhythm in Parkinson’s disease
Source: NPJ Parkinsons Dis. 2024 Mar 5;10:51. doi: 10.1038/s41531-024-00669-3 (PMC10915140; doi:10.1038/s41531-024-00669-3)
Supplement: Supplementary file 1 — Supplementary material [file 41531_2024_669_MOESM1_ESM.pdf]

# Supplementary material: Oscillatory and non-oscillatory features of the magnetoencephalic sensorimotor rhythm in Parkinson's disease

Mikkel C. Vinding* [1,2], Josefine Waldthaler [1,3,4], Allison Eriksson [1,5], Cassia Low Manting [1,6,7], Daniel Ferreira [8,9,10], Martin Ingvar [1], Per Svenningsson [3], Daniel Lundqvist [1]

**Affiliations**

1. NatMEG, Department of Clinical Neuroscience, Karolinska Institutet, Sweden.
2. Danish Research Centre for Magnetic Resonance, Centre for Functional and Diagnostic Imaging and Research, Copenhagen University Hospital - Amager and Hvidovre, Copenhagen, Denmark
3. Section of Neurology, Department of Clinical Neuroscience, Karolinska Institutet, Sweden.
4. Department of Neurology, University Hospital Marburg, Marburg, Germany.
5. Department of Women's and Children's Health, Uppsala University, Sweden.
6. Cognitive Neuroimaging Centre, Lee Kong Chien School of Medicine, Nanyang Technological University, Singapore.
7. McGovern Institute of Brain Research, Massachusetts Institute of Technology, Cambridge, MA, 02139, USA.
8. Division of Clinical Geriatrics, Center for Alzheimer's Research, Department of Neurobiology, Care Sciences, and Society, Karolinska Institutet, Stockholm, Sweden.
9. Department of Radiology, Mayo Clinic, Rochester, MN, USA.
10. Facultad de Ciencias de la Salud, Universidad Fernando Pessoa Canarias, Las Palmas de Gran Canaria, España

## Supplementary Tables

*Regression coefficients for PSD features (1/f corrected)*. Regression coefficients and 95% CI for the regression models of the PSD signal features after removing the 1/f component with Group, Age, Sex, and Cortical Thickness, all two-way interactions, and Age squared. Values with an asterisk (*)indicate factors with Bayes Factor > 3 in the model comparison (see main text).

**1/f offset**

| Variable | Coef | 95%CI | R-hat | P-value | Model comparison (BF) |
|---|---|---|---|---|---|
| Intercept | -1.287 | [-1.419;-1.153] | 1.00 | | |
| Group | 0.309 | [0.137;0.482] | 1.00 | 0.001 | 55.66* |
| Age | 0.007 | [-0.004;0.019] | 1.00 | 0.237 | 0.018 |
| Sex | 0.055 | [-0.109;0.220] | 1.00 | 0.514 | 0.179 |
| Cortical Thickness | -0.042 | [-0.153;0.069] | 1.00 | 0.459 | 0.073 |
| Age2 | -0.001 | [-0.001;0] | 1.00 | 0.062 | 0.009 |
| Group:Age | -0.005 | [-0.017;0.007] | 1.00 | 0.438 | 0.030 |
| Group:Sex | -0.171 | [-0.400;0.055] | 1.00 | 0.143 | 1.32 |
| Group: Cortical Thickness | 0.031 | [-0.085;0.145] | 1.00 | 0.602 | 0.163 |
| Age:Sex | -0.006 | [-0.018;0.007] | 1.00 | 0.421 | 0.033 |
| Age:Cortical Thickness | 0.002 | [-0.005;0.009] | 1.00 | 0.535 | 0.012 |
| Sex:Cortical Thickness | 0.023 | [-0.096;0.142] | 1.00 | 0.711 | 0.163 |

**1/f exponent**

| Variable | Coef | 95%CI | R-hat | P-value | Model comparison (BF) |
|---|---|---|---|---|---|
| Intercept | 0.658 | [0.600;0.715] | 1.00 | | |
| Group | 0.062 | [-0.011;0.137] | 1.00 | 0.101 | 3.92* |
| Age | 0.002 | [-0.003;0.006] | 1.00 | 0.535 | 0.019 |
| Sex | 0.014 | [-0.058;0.086] | 1.00 | 0.706 | 0.106 |
| Cortical Thickness | -0.031 | [-0.080;0.017] | 1.00 | 0.216 | 0.557 |
| Age2 | 0.000 | [0;0] | 1.00 | 0.540 | 4.08e-04 |
| Group:Age | 0.003 | [-0.002;0.008] | 1.00 | 0.272 | 0.009 |
| Group:Sex | 0.001 | [-0.099;0.100] | 1.00 | 0.985 | 0.126 |
| Group: Cortical Thickness | 0.020 | [-0.030;0.071] | 1.00 | 0.438 | 0.091 |
| Age:Sex | -0.003 | [-0.008;0.002] | 1.00 | 0.314 | 0.009 |
| Age:Cortical Thickness | -0.001 | [-0.004;0.001] | 1.00 | 0.403 | 0.006 |
| Sex:Cortical Thickness | -0.012 | [-0.064;0.040] | 1.00 | 0.640 | 0.074 |

**Beta power**

| Variable | Coef | 95%CI | R-hat | P-value | Model comparison (BF) |
|---|---|---|---|---|---|
| Intercept | 0.291 | [0.248;0.332] | 1.00 | | |
| Group | 0.066 | [0.011;0.120] | 1.00 | 0.016 | 0.630 |
| Age | 0.000 | [-0.003;0.004] | 1.00 | 0.817 | 0.017 |
| Sex | -0.008 | [-0.060;0.045] | 1.00 | 0.770 | 0.122 |
| Cortical Thickness | -0.014 | [-0.049;0.021] | 1.00 | 0.428 | 0.098 |
| Age2 | 0.000 | [0;0] | 1.00 | 0.955 | 2.75e-04 |
| Group:Age | 0.004 | [0;0.007] | 1.00 | 0.064 | 0.021 |
| Group:Sex | -0.045 | [-0.116;0.027] | 1.00 | 0.221 | 0.208 |
| Group: Cortical Thickness | -0.003 | [-0.040;0.033] | 1.00 | 0.854 | 0.046 |
| Age:Sex | -0.002 | [-0.006;0.002] | 1.00 | 0.369 | 0.008 |
| Age:Cortical Thickness | 0.000 | [-0.002;0.002] | 1.00 | 0.956 | 0.003 |
| Sex:Cortical Thickness | 0.003 | [-0.034;0.041] | 1.00 | 0.861 | 0.049 |

**Beta centre frequency**

| Variable | Coef | 95%CI | R-hat | P-value | Model comparison (BF) |
|---|---|---|---|---|---|
| Intercept | 21.67 | [20.07;23.29] | 1.00 | | |
| Group | -1.050 | [-3.11;1.001] | 1.00 | 0.317 | 3.06* |
| Age | -0.079 | [-0.225;0.069] | 1.00 | 0.286 | 0.562 |
| Sex | -1.161 | [-3.180;0.851] | 1.00 | 0.257 | 3.02* |
| Cortical Thickness | 0.348 | [-1.00;1.70] | 1.00 | 0.607 | 1.76 |
| Age2 | -0.003 | [-0.011;0.004] | 1.00 | 0.436 | 0.015 |
| Group:Age | -0.125 | [-0.280;0.029] | 1.00 | 0.112 | 0.371 |
| Group:Sex | 1.221 | [-1.56;4.023] | 1.00 | 0.391 | 5.85* |
| Group: Cortical Thickness | -0.181 | [-1.570;1.212] | 1.00 | 0.798 | 1.92 |
| Age:Sex | 0.112 | [-0.052;0.279] | 1.00 | 0.184 | 0.268 |
| Age:Cortical Thickness | 0.066 | [-0.023;0.155] | 1.00 | 0.153 | 0.366 |
| Sex:Cortical Thickness | 0.140 | [-1.298;1.598] | 1.00 | 0.859 | 1.87 |

**Alpha power**

| Variable | Coef | 95%CI | R-hat | P-value | Model comparison (BF) |
|---|---|---|---|---|---|
| Intercept | 0.287 | [0.225;0.349] | 1.00 | | |
| Group | 0.038 | [-0.041;0.117] | 1.00 | 0.347 | 0.166 |
| Age | 0.001 | [-0.004;0.006] | 1.00 | 0.766 | 0.004 |
| Sex | -0.040 | [-0.116;0.037] | 1.00 | 0.300 | 0.156 |
| Cortical Thickness | 0.014 | [-0.038;0.067] | 1.00 | 0.600 | 0.036 |
| Age2 | 0.000 | [0;0] | 1.00 | 0.980 | 4.19e-04 |
| Group:Age | 0.001 | [-0.005;0.006] | 1.00 | 0.884 | 0.008 |
| Group:Sex | -0.006 | [-0.113;0.102] | 1.00 | 0.908 | 0.142 |
| Group: Cortical Thickness | -0.048 | [-0.101;0.006] | 1.00 | 0.084 | 0.284 |
| Age:Sex | -0.001 | [-0.007;0.005] | 1.00 | 0.791 | 0.007 |
| Age:Cortical Thickness | -0.001 | [-0.004;0.002] | 1.00 | 0.617 | 0.005 |
| Sex:Cortical Thickness | 0.013 | [-0.043;0.070] | 1.00 | 0.642 | 0.080 |

**Alpha centre frequency**

| Variable | Coef | 95%CI | R-hat | P-value | Model comparison (BF) |
|---|---|---|---|---|---|
| Intercept | 10.41 | [9.70;11.12] | 1.00 | | |
| Group | -0.569 | [-1.484;0.360] | 1.00 | 0.219 | 1.43 |
| Age | 0.053 | [-0.014;0.120] | 1.00 | 0.119 | 0.106 |
| Sex | -0.186 | [-1.069;0.702] | 1.00 | 0.666 | 0.752 |
| Cortical Thickness | 0.434 | [-0.184;1.058] | 1.00 | 0.172 | 2.00 |
| Age2 | 0.003 | [-0.001;0.006] | 1.00 | 0.174 | 0.012 |
| Group:Age | -0.102 | [-0.172;-0.030] | 1.00 | 0.005 | 1.15 |
| Group:Sex | 0.268 | [-0.964;1.507] | 1.00 | 0.658 | 2.57 |
| Group: Cortical Thickness | -0.618 | [-1.245;0.006] | 1.00 | 0.053 | 6.50* |
| Age:Sex | 0.010 | [-0.062;0.083] | 1.00 | 0.773 | 0.094 |
| Age:Cortical Thickness | 0.026 | [-0.014;0.066] | 1.00 | 0.196 | 0.149 |
| Sex:Cortical Thickness | 0.250 | [-0.406;0.905] | 1.00 | 0.448 | 1.12 |

*Regression coefficients for PSD features without 1/f correction.* Regression coefficients and 95% CI for the regression models of the PSD signal features without removing the 1/f component with Group, Age, Sex, and Cortical Thickness, all two-way interactions, and Age squared. Values with an asterisk (*) indicate factors with Bayes Factor > 3 in the model comparison (see main text).

**Beta power without removal of the 1/f-component**

| **Variable** | **Coef** | **95%CI** | **R-hat** | **P-value** | **Model comparison (BF)** |
|---|---|---|---|---|---|
| Intercept | 2.26E-02 | [0.012;0.032] | 1.00 | | |
| Group | 2.00E-02 | [0.007;0.032] | 1.00 | 0.002 | 0.173 |
| Age | 2.74E-04 | [0;0.001] | 1.00 | 0.546 | 0.001 |
| Sex | -2.80E-04 | [-0.012;0.012] | 1.00 | 0.961 | 0.110 |
| Cortical Thickness | -2.12E-04 | [-0.008;0.008] | 1.00 | 0.961 | 0.005 |
| Age2 | -3.11E-05 | [0;0] | 1.00 | 0.222 | 0.001 |
| Group:Age | -3.59E-04 | [-0.001;0] | 1.00 | 0.460 | 0.002 |
| Group:Sex | -1.50E-02 | [-0.032;0.002] | 1.00 | 0.085 | 0.106 |
| Group: Cortical Thickness | -4.26E-04 | [-0.009;0.008] | 1.00 | 0.920 | 0.011 |
| Age:Sex | 7.60E-05 | [0;0.001] | 1.00 | 0.885 | 0.001 |
| Age:Cortical Thickness | 2.48E-04 | [0;0] | 1.00 | 0.377 | 0.001 |
| Sex:Cortical Thickness | 6.04E-04 | [-0.008;0.009] | 1.00 | 0.897 | 0.011 |

**Beta centre frequency without removal of the 1/f-component**

| **Variable** | **Coef** | **95%CI** | **R-hat** | **P-value** | **Model comparison (BF)** |
|---|---|---|---|---|---|
| Intercept | 17.78 | [16.35;19.22] | 1.00 | | |
| Group | -0.557 | [-2.414;1.282] | 1.00 | 0.557 | 10.95* |
| Age | 0.038 | [-0.093;0.17] | 1.00 | 0.570 | 0.084 |
| Sex | -0.387 | [-2.18;1.407] | 1.00 | 0.669 | 6.863* |
| Cortical Thickness | 0.946 | [-0.264;2.162] | 1.00 | 0.125 | 1.015 |
| Age2 | -0.002 | [-0.009;0.005] | 1.00 | 0.603 | 0.010 |
| Group:Age | 0.0356 | [-0.101;0.174] | 1.00 | 0.607 | 0.177 |
| Group:Sex | -0.928 | [-3.402;1.546] | 1.00 | 0.462 | 3.455* |
| Group: Cortical Thickness | -0.925 | [-2.183;0.339] | 1.00 | 0.147 | 4.341* |
| Age:Sex | -0.115 | [-0.261;0.031] | 1.00 | 0.120 | 0.423 |
| Age:Cortical Thickness | -0.028 | [-0.107;0.051] | 1.00 | 0.492 | 0.132 |
| Sex:Cortical Thickness | -0.202 | [-1.504;1.105] | 1.00 | 0.757 | 1.724 |

**Alpha power without removal of the 1/f-component**

| Variable | Coef | 95%CI | R-hat | P-value | Model comparison (BF) |
|---|---|---|---|---|---|
| Intercept | 0.032 | [0.017;0.046] | 1.00 | | |
| Group | 0.021 | [0.001;0.039] | 1.00 | 0.031 | 0.057 |
| Age | 0.0003 | [0;0.001] | 1.00 | 0.552 | 0.001 |
| Sex | -0.001 | [-0.019;0.017] | 1.00 | 0.919 | 0.084 |
| Cortical Thickness | 0.006 | [-0.006;0.018] | 1.00 | 0.357 | 0.009 |
| Age2 | -4.57E-05 | [0;0] | 1.00 | 0.227 | 0.001 |
| Group:Age | -0.001 | [-0.002;0.000] | 1.00 | 0.299 | 0.003 |
| Group:Sex | -0.016 | [-0.040;0.009] | 1.00 | 0.218 | 0.056 |
| Group: Cortical Thickness | -0.005 | [-0.018;0.007] | 1.00 | 0.424 | 0.026 |
| Age:Sex | 0.0001 | [-0.001;0.001] | 1.00 | 0.832 | 0.002 |
| Age:Cortical Thickness | 0.0004 | [0;0.001] | 1.00 | 0.382 | 0.001 |
| Sex:Cortical Thickness | -0.003 | [-0.015;0.010] | 1.00 | 0.691 | 0.018 |

**Alpha centre frequency without removal of the 1/f-component**

| Variable | Coef | 95%CI | R-hat | P-value | Model comparison (BF) |
|---|---|---|---|---|---|
| Intercept | 9.94 | [9.47;10.43] | 1.00 | | |
| Group | 0.219 | [-0.403;0.837] | 1.00 | 0.483 | 1.431 |
| Age | -0.007 | [-0.050;0.036] | 1.00 | 0.745 | 0.105 |
| Sex | -0.042 | [-0.639;0.551] | 1.00 | 0.889 | 0.753 |
| Cortical Thickness | -0.028 | [-0.430;0.374] | 1.00 | 0.899 | 2.006 |
| Age2 | 0.0003 | [-0.002;0.002] | 1.00 | 0.756 | 0.012 |
| Group:Age | -0.035 | [-0.080;0.011] | 1.00 | 0.140 | 1.145 |
| Group:Sex | -0.207 | [-1.027;0.623] | 1.00 | 0.616 | 2.570 |
| Group: Cortical Thickness | -0.045 | [-0.465;0.372] | 1.00 | 0.838 | 6.515* |
| Age:Sex | 0.017 | [-0.031;0.066] | 1.00 | 0.476 | 0.094 |
| Age:Cortical Thickness | -0.001 | [-0.027;0.025] | 1.00 | 0.940 | 0.149 |
| Sex:Cortical Thickness | 0.156 | [-0.277;0.586] | 1.00 | 0.477 | 1.115 |

*Regression coefficients for burst features.* Regression coefficients and 95% CI for the regression models of the burst features with Group, Age, Sex, and Cortical Thickness, all two-way interactions, and Age squared. Values with an asterisk (*) indicate factors with Bayes Factor > 3 in the model comparison (see main text).

**Burst rate**

| Variable | Coef | 95%CI | R-hat | P-value | Model comparison (BF) |
|---|---|---|---|---|---|
| Intercept | 3.604 | [3.533;3.673] | 1.00 | | |
| Group | 0.003 | [-0.086;0.091] | 1.00 | 0.947 | 0.109 |
| Age | 0.005 | [-0.001;0.011] | 1.00 | 0.152 | 0.052 |
| Sex | -0.146 | [-0.234;-0.056] | 1.00 | 0.002 | 3.80* |
| Cortical Thickness | -0.072 | [-0.130;-0.013] | 1.00 | 0.014 | 0.079 |
| Age2 | 0.000 | [0;0] | 1.00 | 0.599 | 4.98e-04 |
| Group:Age | -0.011 | [-0.017;-0.004] | 1.00 | 0.001 | 3.34* |
| Group:Sex | 0.077 | [-0.044;0.200] | 1.00 | 0.213 | 0.293 |
| Group: Cortical Thickness | 0.011 | [-0.052;0.074] | 1.00 | 0.712 | 0.082 |
| Age:Sex | -0.004 | [-0.011;0.002] | 1.00 | 0.256 | 0.146 |
| Age:Cortical Thickness | 0.004 | [0;0.007] | 1.00 | 0.038 | 0.106 |
| Sex:Cortical Thickness | 0.066 | [0.002;0.129] | 1.00 | 0.043 | 0.633 |

**Burst duration**

| Variable | Coef | 95%CI | R-hat | P-value | Model comparison (BF) |
|---|---|---|---|---|---|
| Intercept | -3.399 | [-3.482;-3.316] | 1.00 | | |
| Group | 0.005 | [-0.101;0.111] | 1.00 | 0.922 | 0.102 |
| Age | -0.002 | [-0.009;0.005] | 1.00 | 0.606 | 0.214 |
| Sex | -0.106 | [-0.210;-0.001] | 1.00 | 0.044 | 0.701 |
| Cortical Thickness | -0.010 | [-0.076;0.057] | 1.00 | 0.777 | 0.042 |
| Age2 | 0.000 | [0;0] | 1.00 | 0.990 | 5.22e-04 |
| Group:Age | -0.005 | [-0.012;0.002] | 1.00 | 0.207 | 0.020 |
| Group:Sex | 0.062 | [-0.080;0.205] | 1.00 | 0.387 | 0.232 |
| Group: Cortical Thickness | 0.012 | [-0.058;0.082] | 1.00 | 0.728 | 0.088 |
| Age:Sex | -0.001 | [-0.009;0.007] | 1.00 | 0.794 | 0.018 |
| Age:Cortical Thickness | 0.004 | [0;0.008] | 1.00 | 0.048 | 0.036 |
| Sex:Cortical Thickness | -0.014 | [-0.085;0.058] | 1.00 | 0.700 | 0.102 |

**Burst interval**

| Variable | Coef | 95%CI | R-hat | P-value | Model comparison (BF) |
|---|---|---|---|---|---|
| Intercept | -0.265 | [-0.413;-0.116] | 1.00 | | |
| Group | -0.068 | [-0.261;0.125] | 1.00 | 0.493 | 0.332 |
| Age | -0.001 | [-0.015;0.012] | 1.00 | 0.808 | 0.210 |
| Sex | 0.154 | [-0.033;0.345] | 1.00 | 0.108 | 0.436 |
| Cortical Thickness | 0.080 | [-0.043;0.201] | 1.00 | 0.211 | 0.146 |
| Age2 | 0.000 | [0;0] | 1.00 | 0.838 | 9.85e-04 |
| Group:Age | 0.009 | [-0.005;0.022] | 1.00 | 0.240 | 0.051 |
| Group:Sex | -0.074 | [-0.332;0.187] | 1.00 | 0.574 | 0.345 |
| Group: Cortical Thickness | -0.013 | [-0.140;0.115] | 1.00 | 0.868 | 0.164 |
| Age:Sex | 0.010 | [-0.005;0.025] | 1.00 | 0.177 | 0.145 |
| Age:Cortical Thickness | -0.004 | [-0.012;0.003] | 1.00 | 0.273 | 0.022 |
| Sex:Cortical Thickness | -0.044 | [-0.176;0.086] | 1.00 | 0.517 | 0.191 |

**Burst amplitude**

| Variable | Coef | 95%CI | R-hat | P-value | Model comparison (BF) |
|---|---|---|---|---|---|
| Intercept | 0.629 | [0.495;0.762] | 1.00 | | |
| Group | 0.361 | [0.181;0.538] | 1.00 | <0.001 | 32.34* |
| Age | 0.006 | [-0.006;0.018] | 1.00 | 0.335 | 0.013 |
| Sex | 0.044 | [-0.124;0.212] | 1.00 | 0.580 | 0.564 |
| Cortical Thickness | -0.016 | [-0.128;0.096] | 1.00 | 0.802 | 0.100 |
| Age2 | -0.001 | [-0.001;0] | 1.00 | 0.109 | 0.007 |
| Group:Age | -0.005 | [-0.018;0.008] | 1.00 | 0.486 | 0.024 |
| Group:Sex | -0.244 | [-0.479;-0.006] | 1.00 | 0.045 | 3.22* |
| Group: Cortical Thickness | 0.000 | [-0.115;0.118] | 1.00 | 0.973 | 0.141 |
| Age:Sex | -0.004 | [-0.017;0.009] | 1.00 | 0.532 | 0.029 |
| Age:Cortical Thickness | 0.004 | [-0.003;0.011] | 1.00 | 0.342 | 0.020 |
| Sex:Cortical Thickness | 0.042 | [-0.074;0.159] | 1.00 | 0.511 | 0.210 |

*Regression coefficients for clinical scores.* Standardised regression coefficients [95%-confidence interval] for the regression models on motor symptoms measured with the MDS-UPDRS-III and its subscales {Citation}. Values in red indicate factors with Bayes Factor > 3 in the model comparison (see next Supplementary Table).

| Variable | MDS-UPDRS III | Midline function | Rest tremor | Rigidity | Bradykinesia right hand | Bradykinesia left hand | Postural and kinetic tremor | Lower limb bradykinesia |
|---|---|---|---|---|---|---|---|---|
| *Intercept* | -0.437 [-0.867;0.026] | -0.408 [-0.813;-0.011] | -0.595 [-0.937;-0.236] | -0.369 [-0.824;0.08] | -0.169 [-0.573;0.205] | 0.145 [-0.293;0.611] | -0.225 [-0.652;0.232] | 0.03 [-0.709;0.225] |
| *PSD beta power* | 0.123 [-0.276;0.572] | 0.371 [-0.034;0.739] | 0.263 [-0.107;0.63] | 0.231 [-0.245;0.699] | -0.106 [-0.548;0.314] | -0.42 [-0.823;-0.04] | 0.081 [-0.357;0.555] | -0.317 [-0.367;0.529] |
| *PSD beta centre frequency* | -0.18 [-0.478;0.14] | -0.064 [-0.35;0.249] | -0.196 [-0.484;0.085] | -0.045 [-0.401;0.288] | -0.058 [-0.367;0.236] | -0.279 [-0.625;0.039] | -0.056 [-0.403;0.31] | -0.109 [-0.404;0.29] |
| *PSD alpha power* | 0.072 [-0.343;0.501] | 0.037 [-0.38;0.459] | -0.198 [-0.608;0.197] | -0.055 [-0.546;0.453] | -0.016 [-0.441;0.395] | 0.518 [0.085;0.971] | 0.04 [-0.452;0.531] | 0.154 [-0.458;0.519] |
| *PSD alpha centre frequency* | 0.174 [-0.098;0.453] | -0.047 [-0.295;0.202] | 0.151 [-0.083;0.391] | 0.070 [-0.252;0.397] | 0.216 [-0.050;0.483] | 0.226 [-0.049;0.507] | 0.142 [-0.168;0.439] | 0.149 [-0.176;0.467] |
| *PSD 1/f intercept* | 0.448 [-0.932;1.832] | 0.512 [-0.917;1.845] | 0.968 [-0.206;2.049] | 0.512 [-0.917;1.845] | -1.016 [-2.333;0.151] | 0.216 [-1.047;1.476] | 0.368 [-1.029;1.770] | -0.487 [-0.999;1.802] |
| *PSD 1/f exponent* | -0.241 [-1.009;0.566] | -0.183 [-1.023;0.642] | -0.442 [-1.086;0.219] | -0.183 [-1.023;0.642] | 0.414 [-0.285;1.118] | -0.184 [-0.915;0.599] | 0.076 [-0.696;0.952] | 0.214 [-0.756;0.865] |
| *Burst rate* | -0.091 [-0.572;0.374] | 0.261 [-0.292;0.782] | -0.124 [-0.532;0.278] | 0.261 [-0.292;0.782] | **-0.713 [-1.174;-0.282]** | -0.024 [-0.501;0.456] | -0.153 [-0.659;0.374] | 0.208 [-0.672;0.364] |
| *Median burst length* | 0.183 [-0.173;0.535] | **0.176 [-0.203;0.565]** | -0.166 [-0.482;0.129] | 0.176 [-0.203;0.565] | -0.077 [-0.414;0.231] | 0.277 [-0.078;0.624] | -0.186 [-0.574;0.208] | 0.321 [-0.557;0.178] |
| *Median time between bursts* | 0.145 [-0.283;0.549] | 0.266 [-0.186;0.726] | 0.031 [-0.334;0.432] | 0.266 [-0.186;0.726] | **-0.489 [-0.917;-0.074]** | 0.169 [-0.275;0.645] | -0.042 [-0.47;0.386] | 0.343 [-0.489;0.427] |
| *Median burst amp.* | -0.213 [-1.418;0.927] | -0.383 [-1.48;0.763] | -0.478 [-1.361;0.483] | -0.383 [-1.48;0.763] | 0.997 [0.019;2.043] | -0.073 [-1.133;0.995] | -0.136 [-1.329;1.026] | 0.278 [-1.413;1.036] |
| *Age* | 0.018 [-0.01;0.050] | 0.009 [-0.022;0.040] | -0.003 [-0.03;0.02] | 0.009 [-0.022;0.04] | -0.007 [-0.033;0.023] | 0.029 [-0.002;0.062] | 0 [-0.032;0.032] | 0.013 [-0.034;0.033] |
| *Sex (male - female)* | **0.672 [0.049;1.200]** | **0.626 [0.045;1.286]** | **0.873 [0.369;1.382]** | 0.626 [0.045;1.286] | 0.184 [-0.313;0.749] | -0.291 [-0.947;0.284] | 0.299 [-0.328;0.915] | -0.082 [-0.31;0.906] |
| *Cortical thickness* | -0.183 [-0.464;0.102] | -0.023 [-0.328;0.293] | -0.089 [-0.34;0.145] | -0.023 [-0.328;0.293] | -0.153 [-0.421;0.11] | -0.199 [-0.483;0.071] | -0.198 [-0.509;0.126] | 0.022 [-0.518;0.126] |
| *Levodopa equivalent daily dosage* | -0.141 [-0.429;0.142] | 0.234 [-0.098;0.582] | **0.138 [-0.116;0.401]** | 0.234 [-0.098;0.582] | 0.019 [-0.244;0.297] | -0.158 [-0.466;0.139] | 0.253 [-0.046;0.546] | 0.041 [-0.069;0.580] |
| *Disease duration* | 0.005 [-0.297;0.295] | -0.333 [-0.663;-0.009] | -0.459 [-0.749;-0.197] | -0.333 [-0.663;-0.009] | 0.026 [-0.259;0.327] | 0.099 [-0.205;0.425] | -0.228 [-0.532;0.124] | -0.171 [-0.568;0.109] |
| *Multiple R-squared* | *0.350* | *0.475* | *0.468* | *0.280* | *0.310* | *0.347* | *0.244* | *0.187* |

Bayes factors (BF) for the analysis of signal features on clinical ratings of motor symptoms in the PD group. BFs indicate comparisons for models with and without the factors in the rows in descending order. Red numbers indicate BF above the threshold (BF > 3) for substantial evidence of an effect. Blue numbers indicate BF below the threshold (BF < 1/3) for substantial evidence of no effect. Black numbers indicate BF in the inconclusive range (1/3 < BF < 3).

| **Variable category** | **Variable** | **total MDS-UPDRS III** | **Midline function** | **Rest tremor** | **Rigidity** | **Bradykinesia right upper extremity** | **Bradykinesia left upper extremity** | **Postural and kinetic tremor** | **Bradykinesia lower limb** |
|---|---|---|---|---|---|---|---|---|---|
| PSD | *Beta power* | *0.161* | 1.44 | 0.503 | 0.254 | 0.153 | 1.73 | 0.137 | 0.412 |
| | *Beta centre frequency* | *0.292* | 0.143 | 0.450 | 0.132 | 0.139 | 0.871 | 0.135 | 0.159 |
| | *Alpha power* | *0.135* | 0.129 | 0.248 | 0.130 | 0.126 | 2.68 | 0.128 | 0.161 |
| | *Alpha centre frequency* | *0.351* | 0.138 | 0.338 | 0.145 | 0.671 | 0.663 | 0.221 | 0.223 |
| | *1/f intercept* | *0.173* | 0.246 | 0.811 | 0.178 | 0.701 | 0.135 | 0.150 | 0.167 |
| | *1/f exponent* | *0.164* | 0.373 | 0.389 | 0.143 | 0.289 | 0.146 | 0.129 | 0.148 |
| Burst | *Rate* | *0.139* | 0.128 | 0.160 | 0.247 | **48.48** | 0.127 | 0.158 | 0.186 |
| | *Length* | *0.261* | **3.56** | 0.272 | 0.221 | 0.145 | 0.625 | 0.232 | 0.664 |
| | *Interval* | *0.171* | 0.336 | 0.128 | 0.292 | **4.23** | 0.188 | 0.129 | 0.445 |
| | *Amplitude* | *0.140* | 0.247 | 0.245 | 0.166 | 1.33 | 0.128 | 0.130 | 0.144 |
| Other | *Age* | *0.346* | 2.99 | 0.132 | 0.157 | 0.148 | 1.29 | 0.126 | 0.190 |
| | *Sex* | ***3.79*** | **13.32** | **132.63** | 1.52 | 0.168 | 0.241 | 0.224 | 0.131 |
| | *Disease duration* | *0.126* | 0.151 | 0.271 | 2.03 | 0.128 | 0.269 | 0.624 | 0.131 |
| | *Levodopa equivalent daily dosage* | *0.231* | 0.158 | **198.68** | 0.491 | 0.129 | 0.169 | 0.451 | 0.246 |
| | *Cortical thickness* | *0.370* | 0.310 | 0.176 | 0.128 | 0.283 | 0.432 | 0.351 | 0.128 |

**Total MDS-UPDRS III**

| | Variable | Coef [95%CI] | t | p-value | Log-likelihood |
|---|---|---|---|---|---|
| | Intercept | -0.437 [-0.867;0.026] | -2.054 | 0.046 | Full model: -73.098 |
| *PSD* | 1/f intercept | 0.448 [-0.932;1.832] | 0.692 | 0.493 | -73.417 |
| | 1/f exponent | -0.241 [-1.009;0.566] | -0.629 | 0.532 | -73.362 |
| | Alpha power | 0.072 [-0.343;0.501] | 0.326 | 0.746 | -73.169 |
| | Alpha centre freq. | 0.174 [-0.098;0.453] | 1.247 | 0.219 | -74.123 |
| | Beta power | 0.123 [-0.276;0.572] | 0.604 | 0.549 | -73.341 |
| | Beta centre freq. | -0.180 [-0.478;0.140] | -1.127 | 0.265 | -73.939 |
| *Burst* | Rate | -0.091 [-0.572;0.374] | -0.387 | 0.701 | -73.198 |
| | Length | 0.183 [-0.173;0.535] | 1.050 | 0.299 | -73.828 |
| | Interval | 0.145 [-0.283;0.549] | 0.677 | 0.502 | -73.404 |
| | Amplitude | -0.213 [-1.418;0.927] | -0.394 | 0.696 | -73.202 |
| *Other* | Age | 0.018 [-0.010;0.050] | 1.238 | 0.222 | -74.109 |
| | Sex | 0.672 [0.049;1.200] | 2.316 | 0.025 | -76.502 |
| | Cortical thickness | -0.183 [-0.464;0.102] | -1.278 | 0.207 | -74.175 |
| | Levodopa equivalent daily dosage | -0.141 [-0.429;0.142] | -0.955 | 0.344 | -73.704 |
| | Disease duration | 0.005 [-0.297;0.295] | 0.041 | 0.968 | -73.099 |

**Axial symptoms/midline functions**

| | Variable | Coef [95%CI] | t | p-value | Log-likelihood |
|---|---|---|---|---|---|
| | Intercept | -0.408 [-0.813;-0.011] | -2.062 | 0.045 | Full model: -68.546 |
| *PSD* | 1/f intercept | 0.606 [-0.607;1.792] | 1.005 | 0.320 | -69.217 |
| | 1/f exponent | -0.457 [-1.136;0.297] | -1.283 | 0.206 | -69.631 |
| | Alpha power | 0.037 [-0.38;0.459] | 0.181 | 0.857 | -68.568 |
| | Alpha centre freq. | -0.047 [-0.295;0.202] | -0.362 | 0.719 | -68.634 |
| | Beta power | 0.371 [-0.034;0.739] | 1.943 | 0.058 | -70.981 |
| | Beta centre freq. | -0.064 [-0.35;0.249] | -0.435 | 0.665 | -68.673 |
| *Burst* | Rate | 0.037 [-0.399;0.491] | 0.168 | 0.867 | -68.565 |
| | Length | 0.373 [0.031;0.686] | 2.293 | 0.026 | -71.886 |
| | Interval | 0.244 [-0.14;0.64] | 1.22 | 0.229 | -69.528 |
| | Amplitude | -0.507 [-1.494;0.506] | -1.007 | 0.319 | -69.219 |
| *Other* | Age | 0.031 [0.004;0.06] | 2.229 | 0.030 | -71.713 |
| | Sex | 0.739 [0.211;1.275] | 2.738 | 0.009 | -73.207 |
| | Cortical thickness | -0.155 [-0.408;0.125] | -1.167 | 0.249 | -69.446 |
| | Levodopa equivalent daily dosage | -0.08 [-0.377;0.198] | -0.581 | 0.564 | -68.771 |
| | Disease duration | 0.07 [-0.19;0.363] | 0.523 | 0.603 | -68.729 |

**Rest tremor**

| | Variable | Coef [95%CI] | t | P value | Log-likelihood ratio |
|---|---|---|---|---|---|
| | Intercept | -0.595 [-0.937;-0.236] | -3.169 | 0.003 | Full model: -65.220 |
| *PSD* | 1/f intercept | 0.968 [-0.206;2.049] | 1.692 | 0.097 | -67.083 |
| | 1/f exponent | -0.442 [-1.086;0.219] | -1.309 | 0.197 | -66.348 |
| | Alpha power | -0.198 [-0.608;0.197] | -1.011 | 0.317 | -65.897 |
| | Alpha centre freq. | 0.151 [-0.083;0.391] | 1.222 | 0.228 | -66.205 |
| | Beta power | 0.263 [-0.107;0.63] | 1.453 | 0.153 | -66.604 |
| | Beta centre freq. | -0.196 [-0.484;0.085] | -1.392 | 0.171 | -66.492 |
| *Burst* | Rate | -0.124 [-0.532;0.278] | -0.596 | 0.554 | -65.457 |
| | Length | -0.166 [-0.482;0.129] | -1.077 | 0.287 | -65.988 |
| | Interval | 0.031 [-0.334;0.432] | 0.164 | 0.870 | -65.238 |
| | Amplitude | -0.478 [-1.361;0.483] | -1.001 | 0.322 | -65.885 |
| *Other* | Age | -0.003 [-0.03;0.02] | -0.261 | 0.795 | -65.266 |
| | Sex | 0.873 [0.369;1.382] | 3.409 | 0.001 | -72.179 |
| | Cortical thickness | -0.089 [-0.34;0.145] | -0.706 | 0.483 | -65.552 |
| | Levodopa equivalent daily dosage | 0.138 [-0.116;0.401] | 1.076 | 0.287 | -65.987 |
| | Disease duration | -0.459 [-0.749;-0.197] | -3.518 | 0.001 | -72.583 |

**Rigidity**

| | Variable | Coef [95%CI] | t | p-value | Log-likelihood |
|---|---|---|---|---|---|
| | Intercept | -0.369 [-0.824;0.08] | -1.583 | 0.120 | Full model: -78.973 |
| *PSD* | 1/f intercept | 0.512 [-0.917;1.845] | 0.72 | 0.475 | -79.318 |
| | 1/f exponent | -0.183 [-1.023;0.642] | -0.437 | 0.665 | -79.100 |
| | Alpha power | -0.055 [-0.546;0.453] | -0.227 | 0.822 | -79.007 |
| | Alpha centre freq. | 0.07 [-0.252;0.397] | 0.456 | 0.651 | -79.112 |
| | Beta power | 0.231 [-0.245;0.699] | 1.029 | 0.309 | -79.674 |
| | Beta centre freq. | -0.045 [-0.401;0.288] | -0.258 | 0.798 | -79.017 |
| *Burst* | Rate | 0.261 [-0.292;0.782] | 1.006 | 0.319 | -79.644 |
| | Length | 0.176 [-0.203;0.565] | 0.919 | 0.363 | -79.533 |
| | Interval | 0.266 [-0.186;0.726] | 1.128 | 0.265 | -79.814 |
| | Amplitude | -0.383 [-1.48;0.763] | -0.645 | 0.522 | -79.250 |
| *Other* | Age | 0.009 [-0.022;0.04] | 0.576 | 0.567 | -79.194 |
| | Sex | 0.626 [0.045;1.286] | 1.966 | 0.055 | -81.463 |
| | Cortical thickness | -0.023 [-0.328;0.293] | -0.151 | 0.881 | -78.988 |
| | Levodopa equivalent daily dosage | 0.234 [-0.098;0.582] | 1.44 | 0.156 | -80.333 |
| | Disease duration | -0.333 [-0.663;-0.009] | -2.082 | 0.043 | -81.750 |

**Bradykinesia right upper extremity**

| | Variable | Coef [95%CI] | t | p-value | Log-likelihood |
|---|---|---|---|---|---|
| | Intercept | -0.169 [-0.573;0.205] | -0.826 | 0.413 | Full model: -70.879 |
| *PSD* | 1/f intercept | -1.016 [-2.333;0.151] | -1.622 | 0.111 | -72.595 |
| | 1/f exponent | 0.414 [-0.285;1.118] | 1.12 | 0.268 | -71.709 |
| | Alpha power | -0.016 [-0.441;0.395] | -0.077 | 0.939 | -70.883 |
| | Alpha centre freq. | 0.216 [-0.05;0.483] | 1.601 | 0.116 | -72.552 |
| | Beta power | -0.106 [-0.548;0.314] | -0.539 | 0.592 | -71.074 |
| | Beta centre freq. | -0.058 [-0.367;0.236] | -0.378 | 0.707 | -70.975 |
| *Burst* | Rate | -0.713 [-1.174;-0.282] | -3.127 | 0.003 | -76.832 |
| | Length | -0.077 [-0.414;0.231] | -0.461 | 0.647 | -71.021 |
| | Interval | -0.489 [-0.917;-0.074] | -2.355 | 0.023 | -74.393 |
| | Amplitude | 0.997 [0.019;2.043] | 1.909 | 0.062 | -73.233 |
| *Other* | Age | -0.007 [-0.033;0.023] | -0.486 | 0.629 | -71.037 |
| | Sex | 0.184 [-0.313;0.749] | 0.658 | 0.514 | -71.168 |
| | Cortical thickness | -0.153 [-0.421;0.11] | -1.105 | 0.275 | -71.688 |
| | Levodopa equivalent daily dosage | 0.019 [-0.244;0.297] | 0.141 | 0.889 | -70.893 |
| | Disease duration | 0.026 [-0.259;0.327] | 0.184 | 0.855 | -70.902 |

**Bradykinesia left upper extremity**

| | Variable | Coef [95%CI] | t | p-value | Log-likelihood |
|---|---|---|---|---|---|
| | Intercept | 0.145 [-0.293;0.611] | 0.673 | 0.505 | full model: -73.935 |
| *PSD* | 1/f intercept | 0.216 [-1.047;1.476] | 0.329 | 0.744 | -74.007 |
| | 1/f exponent | -0.184 [-0.915;0.599] | -0.475 | 0.637 | -74.085 |
| | Alpha power | 0.518 [0.085;0.971] | 2.305 | 0.026 | -77.309 |
| | Alpha centre freq. | 0.226 [-0.049;0.507] | 1.595 | 0.117 | -75.595 |
| | Beta power | -0.42 [-0.823;-0.04] | -2.019 | 0.049 | -76.554 |
| | Beta centre freq. | -0.058 [-0.367;0.236] | -0.378 | 0.707 | -70.975 |
| *Burst* | Rate | -0.024 [-0.501;0.456] | -0.102 | 0.919 | -73.942 |
| | Length | 0.277 [-0.078;0.624] | 1.566 | 0.124 | -75.536 |
| | Interval | 0.169 [-0.275;0.645] | 0.778 | 0.441 | -74.338 |
| | Amplitude | -0.073 [-1.133;0.995] | -0.134 | 0.894 | -73.947 |
| *Other* | Age | 0.029 [-0.002;0.062] | 1.9 | 0.064 | -76.265 |
| | Sex | -0.291 [-0.947;0.284] | -0.989 | 0.328 | -74.584 |
| | Cortical thickness | -0.199 [-0.483;0.071] | -1.37 | 0.177 | -75.168 |
| | Levodopa equivalent daily dosage | -0.158 [-0.466;0.139] | -1.07 | 0.290 | -74.693 |
| | Disease duration | 0.099 [-0.205;0.425] | 0.666 | 0.509 | -74.23 |

**Postural and kinetic tremor**

| | Variable | Coef [95%CI] | t | p-value | Log-likelihood |
|---|---|---|---|---|---|
| | Intercept | -0.225 [-0.652;0.232] | -0.956 | 0.344 | full model: -79.600 |
| *PSD* | 1/f intercept | 0.368 [-1.029;1.770] | 0.512 | 0.611 | -79.775 |
| | 1/f exponent | 0.076 [-0.696;0.952] | 0.179 | 0.859 | -79.621 |
| | Alpha power | 0.040 [-0.452;0.531] | 0.164 | 0.870 | -79.618 |
| | Alpha centre freq. | 0.142 [-0.168;0.439] | 0.92 | 0.362 | -80.162 |
| | Beta power | 0.081 [-0.357;0.555] | 0.359 | 0.722 | -79.686 |
| | Beta centre freq. | -0.056 [-0.403;0.31] | -0.321 | 0.750 | -79.669 |
| *Burst* | Rate | -0.153 [-0.659;0.374] | -0.585 | 0.561 | -79.828 |
| | Length | -0.186 [-0.574;0.208] | -0.959 | 0.342 | -80.21 |
| | Interval | -0.042 [-0.47;0.386] | -0.176 | 0.861 | -79.62 |
| | Amplitude | -0.136 [-1.329;1.026] | -0.228 | 0.821 | -79.634 |
| *Other* | Age | 0 [-0.032;0.032] | -0.012 | 0.990 | -79.6 |
| | Sex | 0.299 [-0.328;0.915] | 0.93 | 0.357 | -80.174 |
| | Cortical thickness | -0.198 [-0.509;0.126] | -1.246 | 0.219 | -80.623 |
| | Levodopa equivalent daily dosage | 0.253 [-0.046;0.546] | 1.565 | 0.124 | -81.199 |
| | Disease duration | -0.228 [-0.532;0.124] | -1.394 | 0.170 | -80.875 |

**Bradykinesia lower extremity**

| | Variable | Coef [95%CI] | t | p-value | Log-likelihood |
|---|---|---|---|---|---|
| | Intercept | 0.03 [-0.709;0.225] | 0.124 | 0.902 | full model: -82.035 |
| *PSD* | 1/f intercept | -0.487 [-0.999;1.802] | -0.652 | 0.518 | -82.319 |
| | 1/f exponent | 0.214 [-0.756;0.865] | 0.486 | 0.629 | -82.193 |
| | Alpha power | 0.154 [-0.458;0.519] | 0.603 | 0.550 | -82.278 |
| | Alpha centre freq. | 0.149 [-0.176;0.467] | 0.929 | 0.358 | -82.608 |
| | Beta power | -0.317 [-0.367;0.529] | -1.342 | 0.186 | -83.22 |
| | Beta centre freq. | -0.109 [-0.404;0.29] | -0.591 | 0.557 | -82.268 |
| *Burst* | Rate | 0.208 [-0.672;0.364] | 0.764 | 0.449 | -82.424 |
| | Length | 0.321 [-0.557;0.178] | 1.596 | 0.117 | -83.698 |
| | Interval | 0.343 [-0.489;0.427] | 1.386 | 0.172 | -83.297 |
| | Amplitude | 0.278 [-1.413;1.036] | 0.446 | 0.658 | -82.168 |
| *Other* | Age | 0.013 [-0.034;0.033] | 0.783 | 0.438 | -82.443 |
| | Sex | -0.082 [-0.31;0.906] | -0.247 | 0.806 | -82.076 |
| | Cortical thickness | 0.022 [-0.518;0.126] | 0.134 | 0.894 | -82.047 |
| | Levodopa equivalent daily dosage | 0.041 [-0.069;0.58] | 0.246 | 0.807 | -82.076 |
| | Disease duration | -0.171 [-0.568;0.109] | -1.006 | 0.320 | -82.706 |

*Correlation between variables.* Statistically significant ($p < 0.05$) correlation coefficients between all the main variables. Green colours indicate a positive correlation between variables, and red colours indicate a negative correlation between variables—the intensity of the colour scales with the correlation coefficient.

| Variable category | Variable | PSD: Beta power | PSD: Beta centre frequency | PSD: Alpha power | PSD: Alpha centre frequency | PSD: 1/f intercept | PSD: 1/f exponent | Burst: Rate | Burst: Duration | Burst: Inteval | Burst: Amplitude | Other: Age | Other: Cortical thickness | Other: MoCA | UPDRS: Midline function | UPDRS: Rest tremor | UPDRS: Rigidity | UPDRS: Bradykinesia upper. | UPDRS: Post./kinetic tremors | UPDRS: Bradykinesia lower. |
|---|---|---|---|---|---|---|---|---|---|---|---|---|---|---|---|---|---|---|---|---|
| PSD | Beta power | | -0,35 | 0,63 | -0,32 | 0,52 | 0,57 | 0,57 | 0,26 | 0,2 | -0,24 | 0,52 | 0,18 | | -0,21 | 0,28 | | | | 0,24 |
| | Beta centre frequency | -0,35 | | -0,34 | | 0,22 | -0,18 | -0,3 | | | | | -0,17 | | | | | | | |
| | Alpha power | 0,63 | -0,34 | | -0,36 | 0,41 | 0,4 | 0,32 | | -0,23 | 0,45 | | | -0,2 | | | | | | |
| | Alpha centre frequency | -0,32 | 0,22 | -0,36 | | -0,19 | -0,43 | | | | | | 0,18 | | | | | | | |
| | 1/f intercept | 0,52 | -0,18 | 0,41 | -0,19 | | 0,56 | 0,55 | 0,31 | -0,48 | 0,91 | | | -0,21 | | | | | | |
| | 1/f exponent | 0,57 | -0,3 | 0,4 | -0,43 | 0,56 | | | | | 0,3 | 0,18 | -0,25 | -0,19 | | | | | 0,25 | |
| Burst | Rate | 0,26 | | 0,32 | | 0,55 | | | 0,55 | -0,8 | 0,55 | | | | | | | -0,23 | | |
| | Duration | 0,2 | | | | 0,31 | | 0,55 | | -0,61 | 0,29 | | | | | | | | | |
| | Inteval | -0,24 | | -0,23 | | -0,48 | | -0,8 | -0,61 | | -0,44 | 0,2 | | | | | | | | |
| | Amplitude | 0,52 | | 0,45 | | 0,91 | 0,3 | 0,55 | 0,29 | -0,44 | | | | -0,21 | | | | | | |
| Other | Age | 0,18 | -0,17 | | | | 0,18 | | | 0,2 | | | -0,19 | -0,22 | 0,37 | | | 0,26 | | |
| | Cortical thickness | | | | 0,18 | | -0,25 | | | | | -0,19 | | 0,19 | -0,38 | | | | | |
| | MoCA | -0,21 | | -0,2 | | -0,21 | -0,19 | | | | -0,21 | -0,22 | 0,19 | | -0,37 | | | -0,26 | -0,23 | |
| UPDRS | Midline function | 0,28 | | | | | | | | | | 0,37 | -0,38 | -0,37 | | 0,38 | 0,38 | 0,4 | 0,28 | 0,32 |
| | Rest tremor | | | | | | | | | | | | | | 0,38 | | 0,37 | 0,28 | 0,53 | 0,23 |
| | Rigidity | | | | | | | | | | | | | | 0,38 | 0,37 | | | | 0,25 |
| | Bradykinesia upper extremity | | | | | | | -0,23 | | | | 0,26 | | -0,26 | 0,4 | 0,28 | | | 0,32 | 0,49 |
| | Postural and kinetic tremors | 0,24 | | | | | 0,25 | | | | | | | -0,23 | 0,28 | 0,53 | | 0,32 | | |
| | Bradykinesia lower limb | | | | | | | | | | | | | | 0,32 | 0,23 | 0,25 | 0,49 | | |
