# Supplementary material for: Oscillatory and non-oscillatory features of the magnetoencephalic sensorimotor rhythm in Parkinson’s disease
Source: NPJ Parkinsons Dis. 2024 Mar 5;10:51. doi: 10.1038/s41531-024-00669-3 (PMC10915140; doi:10.1038/s41531-024-00669-3)
Supplement: Supplementary file 2 — Reporting Summary [file 41531_2024_669_MOESM2_ESM.pdf]

nature portfolio

Corresponding author(s): M.C. Vinding

Last updated by author(s): Dec 7, 2023

Reporting Summary

Nature Portfolio wishes to improve the reproducibility of the work that we publish. This form provides structure for consistency and transparency in reporting. For further information on Nature Portfolio policies, see our Editorial Policies and the Editorial Policy Checklist.

Statistics

For all statistical analyses, confirm that the following items are present in the figure legend, table legend, main text, or Methods section.

n/a	Confirmed	
☐	☒	The exact sample size (*n*) for each experimental group/condition, given as a discrete number and unit of measurement
☐	☒	A statement on whether measurements were taken from distinct samples or whether the same sample was measured repeatedly
☐	☒	The statistical test(s) used AND whether they are one- or two-sided<br>*Only common tests should be described solely by name; describe more complex techniques in the Methods section.*
☐	☒	A description of all covariates tested
☐	☒	A description of any assumptions or corrections, such as tests of normality and adjustment for multiple comparisons
☐	☒	A full description of the statistical parameters including central tendency (e.g. means) or other basic estimates (e.g. regression coefficient) AND variation (e.g. standard deviation) or associated estimates of uncertainty (e.g. confidence intervals)
☐	☒	For null hypothesis testing, the test statistic (e.g. *F*, *t*, *r*) with confidence intervals, effect sizes, degrees of freedom and *P* value noted<br>*Give P values as exact values whenever suitable.*
☐	☒	For Bayesian analysis, information on the choice of priors and Markov chain Monte Carlo settings
☐	☒	For hierarchical and complex designs, identification of the appropriate level for tests and full reporting of outcomes
☐	☒	Estimates of effect sizes (e.g. Cohen's *d*, Pearson's *r*), indicating how they were calculated

Our web collection on statistics for biologists contains articles on many of the points above.

Software and code

Policy information about availability of computer code

Data collection	MEGIN Neuromag Recorder for MEG data recording.
Data analysis	MATLAB with toolbox FieldTrip. MNE-Python. R.<br>The scripts used to process the data and run the analysis presented in the paper are available at: https://github.com/natmegsweden/PD_beta_bursts2.

For manuscripts utilizing custom algorithms or software that are central to the research but not yet described in published literature, software must be made available to editors and reviewers. We strongly encourage code deposition in a community repository (e.g. GitHub). See the Nature Portfolio guidelines for submitting code & software for further information.

Data

Policy information about availability of data

All manuscripts must include a data availability statement. This statement should provide the following information, where applicable:

- Accession codes, unique identifiers, or web links for publicly available datasets
- A description of any restrictions on data availability
- For clinical datasets or third party data, please ensure that the statement adheres to our policy

The full dataset cannot be made publicly available, as the ethical permits for the study do not allow for open data sharing. We have released parts of the MEG data used in this analysis in anonymised form through the EBRAINS data-sharing platform (Vinding et al. 2023)

Research involving human participants, their data, or biological material

Policy information about studies with human participants or human data. See also policy information about sex, gender (identity/presentation), and sexual orientation and race, ethnicity and racism.

Reporting on sex and gender	We aimed at a balanced sample of male and female, given incident ratios of PD is 2:1 male to female ratio. Sex was analyses as a main predictor in the analyses. Sx differences are reported in Results and discussed in Discussion.
Reporting on race, ethnicity, or other socially relevant groupings	No reporting on race, ethnicity, or other social groups.
Population characteristics	Relevant population characteristics are presented at the beginning of Results and summarized in Table 1
Recruitment	The PD patients were recruited from the Parkinson's Outpatient Clinic, Department of Neurology, Karolinska University Hospital, Stockholm, Sweden. The healthy controls were recruited by advertising or amongst spouses of PD patients.
Ethics oversight	. The study was approved by the regional ethics committee in Stockholm (Etikprövningsnämden Stockholm, DNR 2019-00542)

Note that full information on the approval of the study protocol must also be provided in the manuscript.

Field-specific reporting

Please select the one below that is the best fit for your research. If you are not sure, read the appropriate sections before making your selection.

☒ Life sciences ☐ Behavioural & social sciences ☐ Ecological, evolutionary & environmental sciences

For a reference copy of the document with all sections, see nature.com/documents/nr-reporting-summary-flat.pdf

Life sciences study design

All studies must disclose on these points even when the disclosure is negative.

Sample size	There were no a priori sample size calculation. We had originally aim for 100 participants in each group but ended the study early due to COVID lockdown.
Data exclusions	One participant declined to do the MRI scanning, one participant had a scanner malfunction during MRI acquisition, and 11 participants had their MRI scans cancelled due to the COVID-19 pandemic and were not included in the analysis. In total, two PD patients and 11 healthy controls were excluded from the analysis. MEG data segments were excluded if they contained artefacts, as per standard MEG data processing procedures.
Replication	There were no attempt to replicate the findings in an additional study. This study, among other, tries to replicate a previous study.
Randomization	Participants were not randomly allocated to groups.
Blinding	Participants were not randomly allocated to groups. No blinding possible.

Reporting for specific materials, systems and methods

We require information from authors about some types of materials, experimental systems and methods used in many studies. Here, indicate whether each material, system or method listed is relevant to your study. If you are not sure if a list item applies to your research, read the appropriate section before selecting a response.

Materials & experimental systems

n/a	Involved in the study	
☒	☐	Antibodies
☒	☐	Eukaryotic cell lines
☒	☐	Palaeontology and archaeology
☒	☐	Animals and other organisms
☐	☒	Clinical data
☒	☐	Dual use research of concern
☒	☐	Plants

Methods

n/a	Involved in the study	
☒	☐	ChIP-seq
☒	☐	Flow cytometry
☐	☒	MRI-based neuroimaging

Clinical data

Policy information about clinical studies

All manuscripts should comply with the ICMJE guidelines for publication of clinical research and a completed CONSORT checklist must be included with all submissions.

Clinical trial registration	*Provide the trial registration number from ClinicalTrials.gov or an equivalent agency.*
Study protocol	*Note where the full trial protocol can be accessed OR if not available, explain why.*
Data collection	*Describe the settings and locales of data collection, noting the time periods of recruitment and data collection.*
Outcomes	*Describe how you pre-defined primary and secondary outcome measures and how you assessed these measures.*

Plants

Seed stocks	*Report on the source of all seed stocks or other plant material used. If applicable, state the seed stock centre and catalogue number. If plant specimens were collected from the field, describe the collection location, date and sampling procedures.*
Novel plant genotypes	*Describe the methods by which all novel plant genotypes were produced. This includes those generated by transgenic approaches, gene editing, chemical/radiation-based mutagenesis and hybridization. For transgenic lines, describe the transformation method, the number of independent lines analyzed and the generation upon which experiments were performed. For gene-edited lines, describe the editor used, the endogenous sequence targeted for editing, the targeting guide RNA sequence (if applicable) and how the editor was applied.*
Authentication	*Describe any authentication procedures for each seed stock used or novel genotype generated. Describe any experiments used to assess the effect of a mutation and, where applicable, how potential secondary effects (e.g. second site T-DNA insertions, mosiacism, off-target gene editing) were examined.*

Magnetic resonance imaging

Experimental design

Design type	Structrual MRI for MEG source analysis
Design specifications	Single session
Behavioral performance measures	None

Acquisition

Imaging type(s)	Structural
Field strength	3.0 T
Sequence & imaging parameters	3D T1-weighted magnetisation-prepared rapid gradient-echo (MPRAGE) sequence structural images (voxel size: 1x1x1 mm) were obtained on a GE Discovery 3.0 T MR scanner for morphological analysis and creating source spaces for MEG source reconstruction. Multi-echo "FLASH" images were obtained to create volumetric headmodels for MEG source reconstruction
Area of acquisition	Whole head MRI

Diffusion MRI ☐ Used ☒ Not used

Preprocessing

Preprocessing software	Freesurfer
Normalization	No normalization
Normalization template	*Describe the template used for normalization/transformation, specifying subject space or group standardized space (e.g. original Talairach, MNI305, ICBM152) OR indicate that the data were not normalized.*
Noise and artifact removal	*Describe your procedure(s) for artifact and structured noise removal, specifying motion parameters, tissue signals and physiological signals (heart rate, respiration).*
Volume censoring	*Define your software and/or method and criteria for volume censoring, and state the extent of such censoring.*

Statistical modeling & inference

Model type and settings	We did no statistical tests on MRI data
Effect(s) tested	*Define precise effect in terms of the task or stimulus conditions instead of psychological concepts and indicate whether ANOVA or factorial designs were used.*

Specify type of analysis: ☒ Whole brain ☐ ROI-based ☐ Both

Statistic type for inference (See Eklund et al. 2016)	*Specify voxel-wise or cluster-wise and report all relevant parameters for cluster-wise methods.*
Correction	*Describe the type of correction and how it is obtained for multiple comparisons (e.g. FWE, FDR, permutation or Monte Carlo).*

Models & analysis

n/a	Involved in the study
☒	☐ Functional and/or effective connectivity
☒	☐ Graph analysis
☒	☐ Multivariate modeling or predictive analysis
